# Bacterial biofilms colonizing plastics in estuarine waters, with an emphasis on *Vibrio* spp. and their antibacterial resistance

**DOI:** 10.1371/journal.pone.0237704

**Published:** 2020-08-17

**Authors:** Amanda L. Laverty, Sebastian Primpke, Claudia Lorenz, Gunnar Gerdts, Fred C. Dobbs

**Affiliations:** 1 Department of Ocean, Earth and Atmospheric Sciences, Old Dominion University, Norfolk, Virginia, United States of America; 2 Alfred-Wegener-Institute Helmholtz Centre for Polar and Marine Research, Biologische Anstalt Helgoland, Helgoland, Germany; Loyola University Chicago, UNITED STATES

## Abstract

Since plastics degrade very slowly, they remain in the environment on much longer timescales than most natural organic substrates and provide a novel habitat for colonization by bacterial communities. The spectrum of relationships between plastics and bacteria, however, is little understood. The first objective of this study was to examine plastics as substrates for communities of Bacteria in estuarine surface waters. We used next-generation sequencing of the 16S rRNA gene to characterize communities from plastics collected in the field, and over the course of two colonization experiments, from biofilms that developed on plastic (low-density polyethylene, high-density polyethylene, polypropylene, polycarbonate, polystyrene) and glass substrates placed in the environment. Both field sampling and colonization experiments were conducted in estuarine tributaries of the lower Chesapeake Bay. As a second objective, we concomitantly analyzed biofilms on plastic substrates to ascertain the presence and abundance of *Vibrio* spp. bacteria, then isolated three human pathogens, *V*. *cholerae*, *V*. *parahaemolyticus*, and *V*. *vulnificus*, and determined their antibiotic-resistant profiles. In both components of this study, we compared our results with analyses conducted on paired samples of estuarine water. This research adds to a nascent literature that suggests environmental factors govern the development of bacterial communities on plastics, more so than the characteristics of the plastic substrates themselves. In addition, this study is the first to culture three pathogenic vibrios from plastics in estuaries, reinforcing and expanding upon earlier reports of plastic pollution as a habitat for *Vibrio* species. The antibiotic resistance detected among the isolates, coupled with the longevity of plastics in the aqueous environment, suggests biofilms on plastics have potential to persist and serve as focal points of potential pathogens and horizontal gene transfer.

## Introduction

Plastic pollution is omnipresent in marine and estuarine environments, including all coastal areas and remote beaches [1, 2], throughout the open ocean and water column [3], trapped in sea ice in both the Arctic and Antarctic [4, 5], and on the sea floor [6, 7]. One estimate of plastic standing stock in surface waters of the world’s oceans is, at a minimum, 5.25 trillion plastic particles with a mass of 268,940 tons [3]. This standing stock estimate represents only 0.01–0.1% of the plastic believed to have entered the marine environment annually.

Colonization of marine plastic debris was first documented in the 1970s, when diatoms and other microbes were found on plastics in the Sargasso Sea [8] and other areas in the North Atlantic Ocean [9]. Not until more recently, however, has plastic been more vigorously examined as a habitat for aquatic microbial communities [10–13]. Plastics remain in the environment on drastically longer timescales than most natural organic substrates. These long-lasting, durable, often buoyant, physically and chemically distinct substrates thus provide a novel habitat for the colonization and possible dissemination of microbial communities, including bacteria that are human pathogens [14, 15].

Zettler et al. (2013) showed a high diversity of heterotrophs, autotrophs, predators, and pathogens living in the ‘Plastisphere’, the term they coined to encompass the environment in, on, and immediately surrounding a plastic piece in the marine environment [14]. Their studies showed ‘Plastisphere’ communities differed from those in surrounding seawater [14] and suggested these communities also differ latitudinally and between ocean basins [16]. Additionally, in one sample of polypropylene, they found a member of the bacterial genus *Vibrio* to be a dominant component of the community, suggesting that plastic might serve as a ‘vector’ of pathogens and infectious diseases [14]. Since then, other studies have confirmed the presence of *Vibrio*, as well as other potentially pathogenic bacteria and harmful algae, on marine plastics [13, 15, 17].

*Vibrio* is a ubiquitous bacterial genus with wide-ranging and variable habitat preferences, encompassing both host-associated and free-living representatives: multicellular marine hosts, ambient aquatic environments, natural organic substrates, phytoplankton blooms, and now plastic particles [18]. The genus includes human and animal pathogens that have caused major pandemics and countless epidemics across the globe. These pathogens can also inflict expensive losses on aquaculture enterprises. Given their impact on human and animal health and the relative ease of their culture, vibrios have been well studied [18, 19]. In this regard, different species of *Vibrio* form cohesive groups within which they easily exchange genetic elements to confer greater antibiotic resistance, as well as regulate virulence [20]. The concern with plastic serving as a vector of pathogenic organisms may well be compounded by the potential for dissemination of antibiotic-resistance genes associated with “Plastisphere” biofilm communities.

Here we report plastic (low-density polyethylene (LDPE), high-density polyethylene (HDPE), polypropylene (PP), polycarbonate (PC), and polystyrene (PS)) and glass, hung from a floating dock, and microplastics, collected from the environment, as colonization substrates for bacterial communities, with particular focus on *Vibrio* spp., especially the human pathogens, *V*. *cholerae*, *V*. *parahaemolyticus*, and *V*. *vulnificus*. We determined antibiotic-resistance profiles of these vibrios isolated from biofilms on plastics and glass, as well as from surrounding estuarine water. Finally, we characterized and compared bacterial communities in the biofilms and ambient waters using next-generation sequencing (NGS) of a portion of their 16S rRNA gene.

## Materials and methods

### Environmental samples: Collection and analyses

A zooplankton net (80μm mesh, 30 cm diameter) was towed (100 meter tow length) a total of twenty-five separate times from June through November 2015 to collect environmental samples in surface waters at Old Dominion University’s Sailing Center (university property, permit not required) on the Elizabeth River (36.885830, -76.318514). Water samples were collected into sterile Nalgene containers at the same time. Putative microplastic pieces were sorted using sterile forceps, photographed with a stereo-zoom microscope/camera, and their dimensions recorded. Individual putative microplastic pieces were given a unique ID beginning with “AL” and ending with a number based on order of collection (AL1 –AL51). Textile fibers were excluded from the analysis.

After rinsing with sterile Phosphate-Buffered Saline (PBS), putative microplastic pieces were transferred to separate 15ml Falcon tubes containing sterile PBS and vortexed rigorously for one minute to dislodge components of the biofilm community. Vortex agitation did not remove all the biofilm from the surface, but in a functionally defined and easily reproduced method, yielded a sample of removable organisms. The biofilm suspension was filtered onto a sterile 0.2μm filter. Water samples were filtered and treated using the same protocol. Using sterile forceps, the filter was placed on CHROMagar^TM^ Vibrio medium (CHROMagar, Paris, France), selective for the detection and isolation of *V*. *parahaemolyticus*, *V*. *vulnificus*, and *V*. *cholerae*, then incubated at 35°C. After 24 hours, putative *Vibrio* colonies were quantified and expressed as colony-forming units (CFUs) per square cm of substrate or ml of water. Beginning with sample AL23 and continuing through AL51, the biofilm suspension was divided into half and each half was filtered onto separate, sterile 0.2μm sterile filters. One filter was processed for *Vibrio* spp. as above, the other was placed into a sterile 15ml Falcon tube and frozen at -80°C for subsequent DNA extraction. Putative microplastic pieces for Attenuated Total Reflectance Fourier Transform Infrared spectroscopy (ATR-FTIR) were fixed in 2.5% glutaraldehyde (final conc.) and refrigerated (4°C).

### Environmental samples: ATR-FTIR

A subset (n = 23) of the fixed microplastic samples representing the diversity in shape and color of pieces in the collection was analyzed using ATR-FTIR. Prior to analysis, samples were treated overnight with 1ml H_2_O_2_ (35%, filtered through 0.2μm Anodisc filters) to remove organic matter. Samples then were carefully washed with water (MilliQ, 1ml, two times) and dried at 30°C overnight. ATR-FTIR measurements were performed on a Bruker TENSOR 27 spectrometer, equipped with a Bruker Platinum-Diamant-ATR unit. Measurements were performed in a wavenumber range of 4000–400 cm^-1^, with a resolution of 4 cm^-1^, 32 co-added scans, using Blackman-Harris Term 3 apodization and a zero filling factor of 2 for Fourier-Transformation with the function Power / No peak search for phase correction. For analysis and data collection, the Bruker OPUS 7.5 software was used. The spectra were compared to a database consisting of polymer and biological substances based on vector-normalization. The results were given as a hit value as quality index (highest value, representing the best possible identification, is 1000, the lowest 300).

### Colonization experiments: Design, sampling sequence, and analyses

In October 2015, four plastic types (LDPE, HDPE, PP, PC) and glass substrates were hung from a floating dock in the Lafayette River (36.901649, -76.296715; private home, permit not required), approximately 10 cm below the water surface, to follow *Vibrio* spp. colonization and total community composition over a geometric time series emphasizing the early days (1, 2, 4, 8, 16, and 30 days). Microscope slides were used for the glass substrate. Similar-sized plastic pieces were prepared from Nalgene plasticware purchased from ThermoFisher Scientific and surface sterilized with ethanol prior to emplacement in the river. On each sampling day, three pieces of each plastic type and three water samples were collected, and water temperature and salinity were recorded.

On return to the laboratory, plastic and glass slides were gently rinsed with sterilized PBS to remove non-adherent microorganisms. A section (2.5 x 5.2cm) of each substrate was scraped using sterile inoculation loops and biofilms were transferred to separate 15ml Falcon tubes containing sterile PBS. Falcon tubes were lightly vortexed to ensure a well-mixed solution. Each biofilm suspension was divided in half and filtered onto separate 0.2μm sterile filters. One filter was placed on CHROMagar^TM^ and incubated at 35°C for colony counts of *Vibrio* spp. Log_10_ transformed colony counts were analyzed using a two-way analysis of variance (ANOVA) to assess differences over time and between substrate type. The other filter was placed into a sterile 15ml Falcon tube and frozen at -80°C for subsequent DNA extraction. Corresponding water samples were treated using the same protocol, except that in this first colonization experiment (Colonization Experiment #1), water samples were assayed only for *Vibrio* spp. colony counts, not for bacterial community DNA extraction.

A second colonization experiment (Colonization Experiment #2) was performed beginning in January 2016, employing a similar geometric time series (1, 2, 4, 9, 17, and 31 days), but with one additional type of plastic (PS; slides also prepared from Nalgene plasticware). Biofilms from five plastics and glass, together with samples of water, were collected for colony counts of *Vibrio* spp. and DNA extraction. In all, 183 plastic and glass slides and 33 water samples were collected and analyzed over the course of the two experiments.

### Environmental samples and colonization experiments: PCR identification of *Vibrio* spp.

From the CHROMagar^TM^ plates, 3–5 colonies of putative *Vibrio* spp. were ‘picked’, separately grown overnight in Luria-Bertani (LB) broth + 1% NaCl at 35°C, then 500μl of the bacterial suspension was placed into glycerol (50:50 v/v) and frozen at -80°C for PCR analysis. Isolates were revived in LB broth + 1% NaCl at 35°C for PCR identification. After 24 hours, 400μl of each culture was placed separately into sterile 1.5ml microcentrifuge tubes and centrifuged at 11,290 rcf (Marathon 21000R) for 15 minutes. The supernatant was decanted and 100μl of PBS was added to the tubes. The tubes were held in boiling water for 15 minutes to extract DNA, centrifuged at 11,290 rcf for 10 minutes, and the supernatant pipetted to new 1.5ml microcentrifuge tubes and stored at -20°C until used for PCR analysis for *V*. *cholerae*, *V*. *vulnificus*, or *V*. *parahaemolyticus*.

PCR reactions were run to determine how many of the putative vibrios isolated on CHROMagar^TM^ were indeed *Vibrio* spp. The forward 567 (5′–GGCGTAAAGCGCATGCAGGT– 3′) and the reverse 680 primers (5′–GAAATTCTACCCCCCTCTACAG– 3′) generated a 120 bp long amplicon [21]. The temperature profile for the PCR was as follows: an initial step of 10 min at 94°C, followed by 25 cycles of denaturation for 30 s at 94°C, annealing for 30 s at 55°C and primer extension for 1 min at 72°C. After the 35th cycle, the extension step was prolonged for 7 min to complete synthesis of all strands, and then the samples were kept at 4°C until analysis.

PCR reactions targeted the hemolysin/cytolysin gene *vvhA* of *V*. *vulnificus* and produced a single 411 bp product with the use of primers vvhA-F 5′-AGCGGTGATTTCAACG -3′ and vvhA-R 5′- GGCCGTCTTTGTTCACT-3′ [21]. The thermal cycling conditions for *vvhA* consisted of an initial denaturation step at 94°C for 3 min, followed by 30 cycles of 45 s at 94°C (denaturation), 45 s at 55°C (annealing) and 45 s at 72°C (extension). A final extension step of 2 min at 72°C was performed.

The detection of *V*. *parahaemolyticus* was based on the amplification of the *tlh* gene (thermolabile hemolysin). The forward (5′- ACTCAACACAAGAAGAGATCGACAA-3′) and the reverse primers (5′ GATGAGCGGTTGATGTCCAA-3′) generated a 233 bp long amplicon [22]. Initial denaturation for *tlh* occurred at 95°C for 4 minutes followed by 33 cycles of annealing and extension. Per cycle, the samples were heated to 95°C for 1 minute, cooled to 55°C for 1 minute, and heated to 72°C for 1 minute. In the final extension phase, the samples were held at 72°C for 5 minutes.

*V*. *cholerae* primers Pvc-F *groEL* (5′-GGTTATCGCTGCGGTAGAAG-3′) and Pvc-R *groEL* (5-ATGATGTTGCCCACGCTAGA-3′) produced a 116 bp product [23]. PVC-*groEL* samples called for denaturation at 95°C for 5 minutes, followed by 25 cycles of annealing and extension at 95°C for 5 seconds, 58°C for 30 seconds, and 72°C for 15 seconds. There was no final extension phase.

All reactions were run in either a PTC-100 Peltier Thermal Cycler (BioRad, Hercules, CA) or a Edvocycler (Edvotek, Washington, DC) in a total volume of 25μl composed of 2X Blue-Hot-Start-Taq (Denville Scientific Inc., Metuchen, NJ), 0.1μM of each primer (synthesized by MGW Operon, Huntsville, Alabama), 1% (final concentration) of dimethyl sulfoxide (DMSO, Sigma) and an appropriate volume of sterile MilliQ water to bring the reaction to a final volume of 25μl.

Two microliters of *V*. *vulnificus* FVV DNA, *V*. *parahaemolyticus* 7P, and *V*. *cholerae* O139 were used as positive controls for each specific PCR reaction. No-template controls (master mix without any DNA template), together with positive controls for the two other PCR protocols, were the negative controls for all *Vibrio* species. PCR products were visualized using either a 1.5% or 2.0% agarose gel dyed with ethidium bromide. The gels were viewed and photographed using a Kodak Imaging System (Gel Logic 100) and a UV transilluminator (TFX-35M, Life Technologies).

### Environmental samples and colonization experiments: Antibiotic-resistance testing of *Vibrio* spp.

Isolates (n = 97) from environmental samples and colonization experiments subsequently PCR-confirmed as *V*. *cholerae*, *V*. *vulnificus*, or *V*. *parahaemolyticus* were evaluated for their resistance to a suite of six antibiotics: tetracycline (30μg), chloramphenicol (30μg), gentamicin (10μg), ampicillin (10μg), streptomycin (10μg), and rifampin (5μg). All isolates were tested using the Antimicrobial Susceptibility Testing method of the National Committee for Clinical Laboratory Standards [24].

Isolates were grown on LB + 1% NaCl agar plates to ensure they displayed no contamination before being transferred to LB broth (4.5ml) and incubated at 35°C until the density of the suspension approximately equaled 0.5 McFarland (purchased standard; Becton Dickinson, Inc., New Jersey). A sterile cotton swab was then used to inoculate the Mueller-Hinton agar by dipping into the suspension, removing excess liquid by turning the swab against the side of the tube, and then spreading evenly over the entire plate by rotating the plate 60° three times [25]. Mueller-Hinton agar (Becton Dickinson, Inc., New Jersey) was prepared according to the manufacturer’s requirements and two plates were prepared for each isolate with six antibiotics applied per plate. Antibiotic discs were applied using a Sensi-Disc dispenser (Becton Dickinson, Inc., New Jersey) and plates were incubated for 18–24 hours at 35°C. Any zones of growth inhibition (ZOI) around the antibiotic disks were then measured with a metric ruler and the susceptibility was categorized according to standard ZOI measurements for each antibiotic (i.e. susceptible, intermediate, or resistant). The quality of media, discs, and technique was ensured using *Escherichia coli* as a control organism. Wilcoxon rank-sum tests were used to identify statistically significant differences in antibiotic profiles between isolates from environmental samples and colonization experiments.

ZOI data were analyzed using PRIMER (Plymouth Routines in Multivariate Ecological Research) V6 software (PRIMER-E, Plymouth, UK). Similarity indices based on Euclidean distance were calculated for all pairwise combinations of isolates’ antibiotic susceptibility profiles. Relationships were examined by cluster analysis and demonstrated with plots of principal-component similarity coefficients.

### Environmental samples and colonization experiments: Biofilm DNA extractions, sequencing, and sequence processing

DNA was extracted from frozen filters using MO BIO’s PowerBiofilm® DNA isolation kit. DNA was also extracted from biofilms on four environmental samples confirmed by ATR-FTIR to be polyethylene (PE), and their corresponding water samples. To check the quality of the extracted DNA, a 16S PCR was run using primers BAC-8F (5'-AGAGTTTGATCCTGGCTCAG-3') [26] and 1492R (5′-GGTTACCTTGTTACGACTT-3′) to amplify an approximately 1,500 bp long fragment of bacterial 16S rRNA gene [27]. This PCR consisted of an initial denaturation step at 94°C for 1 min followed by 30 PCR cycles (95°C denaturation for 1 min; primer annealing at 55C°for 1 min; and primer extension at 72°C for 2 min), and a final 7 min elongation step at 72°C. Extraction controls did not yield amplicons. DNA concentration and quality were determined by microspectrophotometry (Nano-Drop ND 2000C).

DNA was shipped to Mr. DNA (www.mrdnalab.com, Shallowater, TX, USA) and sequenced with MiSeq Illumina technology with amplification of the V3-V4 hypervariable region of the 16S rRNA gene. Sequencing was performed on a MiSeq following the manufacturer’s guidelines. The 16S rRNA gene PCR primers 341/806 with barcode on the forward primer were used in a 28 cycle PCR using the HotStarTaq Plus Master Mix Kit (Qiagen, USA) under the following conditions: 94°C for 3 minutes, followed by 28 cycles of 94°C for 30 seconds, 53°C for 40 seconds and 72°C for 1 minute, after which a final elongation step at 72°C for 5 minutes was performed. After amplification, PCR products were checked in a 2% agarose gel to determine the success of amplification. Multiple samples were pooled together based on their molecular weight and DNA concentrations, then purified using calibrated Ampure XP beads. The pooled and purified PCR product was used to prepare the Illumina DNA library.

Sequencing data was analyzed using the MOTHUR pipeline v.1.35.1 [28]. Sequences were depleted of barcodes and primers, then low quality sequences or sequences < 300bp, sequences with ambiguous base calls, and sequences with homopolymer runs exceeding 8 bp were removed [28]. Sequences were subsequently aligned using the SILVA (http://www.mothur.org/wiki/Silva_reference_files) reference database and were then further denoised (http://www.mothur.org/wiki/Pre.cluster) [29]. Chimeras were removed with VSEARCH algorithm implemented in MOTHUR (https://github.com/torognes/vsearch) [30]. High quality sequences were classified (domain to genus level) using the Ribosomal Database Project (RDP) Naïve Bayesian Classifier [31] and contaminants (e.g. Archaea, Eukarya, mitochondria, and unknown domain) were removed (some Eukarya sequences required manual removal during analysis). DNA distance matrices were calculated and used to define the number of operational taxonomic units (OTUs) at sequence divergences of 3% (97% similarity) [32]. To normalize the sequence effort across samples, sequences were randomly subsampled to the sample with the fewest number of reads (2,500 sequences, 2.2.HDPE2). Sample coverage was estimated using Good’s Coverage. Estimated Bacterial diversity richness was calculated using Shannon indices [28]. Cluster analysis on log (x+1) transformed sequence abundances was performed and then the Bray-Curtis indices [33] were calculated at 1,000 bootstrap values, to graphically illustrate the relationships among the different samples.

## Results

### Salinity and temperature at the sampling sites

Environmental samples were collected between June and November of 2015, when water temperatures ranged between 10.3 and 30.1°C and salinity (September to November only) between 17 and 23 ppt (S1 Table). In Colonization Experiment #1, temperature and salinity ranged between 14.8 and 21.5°C and 19 to 22 ppt, respectively. In Colonization Experiment #2, temperature and salinity varied from 2.1 to 9.5°C and 12 to 18 ppt, respectively (S1 Table).

### Environmental samples: Microplastics’ abundance, identification, and *Vibrio* spp. concentration

**Fig 1:**
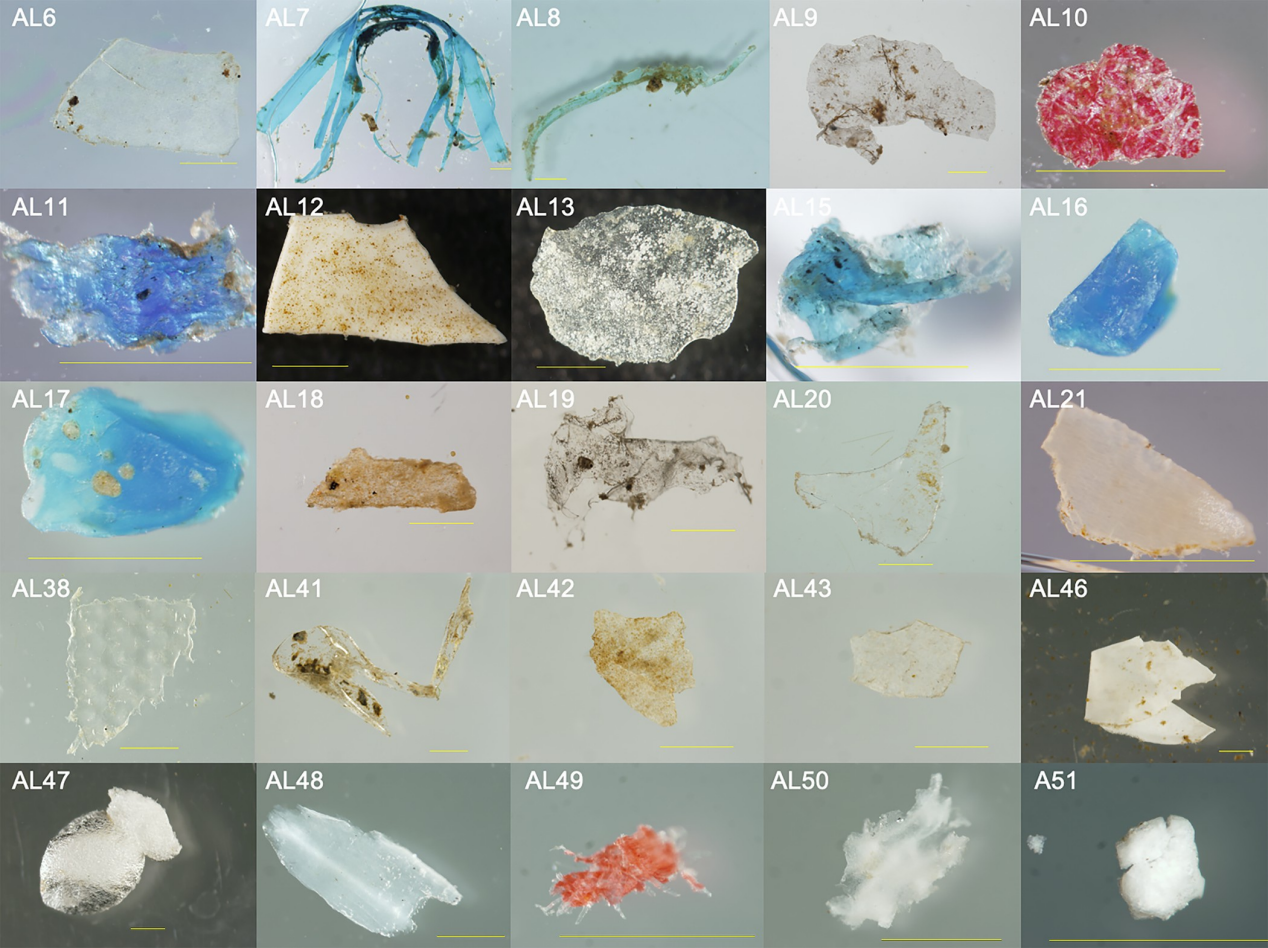
Representative putative microplastics from environmental samples photographed with a stereo-zoom microscope/camera after their collection from surface waters of the Elizabeth River at Old Dominion University’s Sailing Center. Samples were collected from June through November 2015. Most pieces were distinctly biofouled. Bars represent one millimeter.

In total, 51 putative microplastics were collected from approximately 707,000 liters of water sampled (calculated using radius of net and total distance towed), equating to approximately 0.07 pieces/m^3^. Pieces ranged between 0.14mm and 8.62mm in size (longest dimension), with only two pieces surpassing the 5mm literature standard for microplastics. Most pieces were transparent (others were white, yellow, red, and blue) and distinctly biofouled (Fig 1). Of 51 pieces, 23 were selected for examination using ATR-FTIR. Polymer identification was not possible for 6 pieces, as the quality indices necessary for their identification were divided among PE, PP, viscose, or cellulose. Of the 17 pieces successfully identified, the majority were PE (71%), while the rest were PP, PS, wood, p-vinylpyrrolidone/vinyl acetate, or cellulose/viscose (Table 1). Four of the FTIR-confirmed PE plastics were determined to be oxidized or partially oxidized. Biofilm was still present on many samples, even after they had been processed for bacterial culturing and treated overnight with hydrogen peroxide.

**Table 1:** Identification of 23 putative microplastic pieces using ATR-FTIR.

Sample ID	Identification	Biofilm Present?
AL6	No clear ID possible	No
AL7	Polypropylene	Yes
AL8	Polyethylene	Yes
AL9	Polyethylene	Yes
AL10	Wood	No
AL11	Polyethylene	Yes
AL12	No clear ID possible	No
AL13	Polyethylene	Yes
AL15	Polyethylene	Yes
AL16	Polyethylene	No
AL17	Polyethylene	No
AL18	No clear ID possible	No
AL19	Polyethylene	Yes
AL20	No clear ID possible	No
AL21	No clear ID possible	No
AL38	Polyethylene	Yes
AL41	No clear ID possible	Yes
AL42	Polyethylene	Yes
AL43	Polyethylene	Yes
AL46	Polyethylene	Yes
AL47	Polystyrene	No
AL48	P-vinylpyrrolidone/vinyl acetate copolymer	No
AL50	Fabric material; cellulose/viscose	No

**Fig 2:**
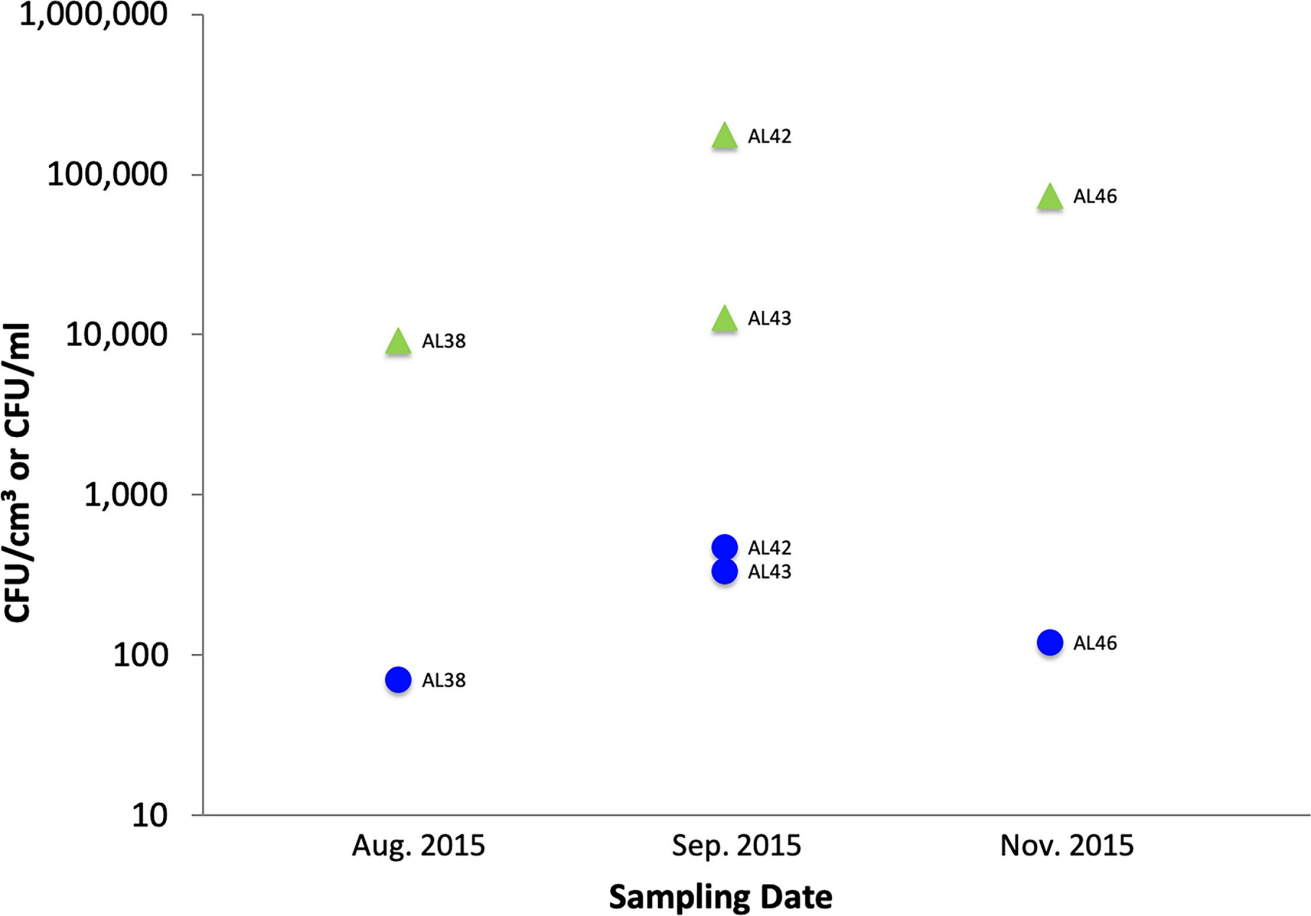
Concentrations of putative *Vibrio* spp. on PE (environmental samples; green triangles) and in paired water samples (filled blue circles).

The concentration of putative *Vibrio* spp. on microplastics was much greater than in corresponding water samples. For example, samples AL38, 42, 43, and 46, all confirmed by ATR-FTIR as PE, exhibited enriched concentrations (CFU/cm^3^) of putative *Vibrio* spp. by at least one to two orders of magnitude compared to concentrations (CFU/ml) in paired water samples (Fig 2). Median values were 43,307 and 225 CFUs, respectively; the distributions in the two groups differed significantly (Wilcoxon ranksum = 26, *n*_1_ = 4 *n*_2_ = 4, p = 0.029, two-tailed).

### Colonization experiments: *Vibrio* spp. concentrations over time and by substrate

**Fig 3:**
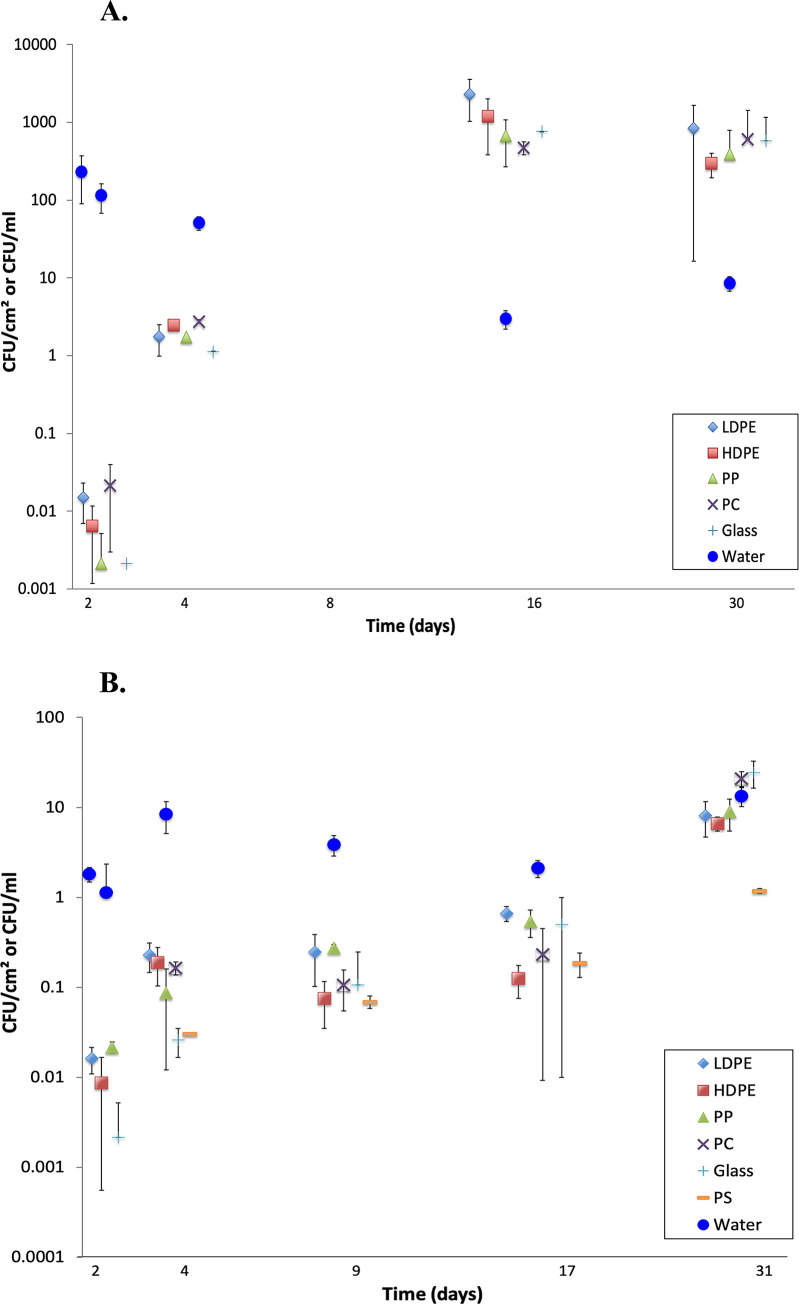
**A.** Colonization Experiment #1 (12 Oct– 10 Nov 2015): Mean (n = 3, ± 1 sd) concentrations of putative *Vibrio* spp. from biofilms on plastics and glass, and from paired water samples. Water temperature ranged from 15–22°C. For clarity, points are jittered about the day of sampling. Values for day 1 all were zero and are not displayed. Substrate types: low-density polyethylene (LDPE), high-density polyethylene (HDPE), polypropylene (PP), polycarbonate (PC), and glass (Glass). **B.** Colonization Experiment #2 (12 Jan– 10 Feb 2016): Mean (+ 1 sd) concentrations of putative *Vibrio* spp. from biofilms on plastics and glass, and from paired water samples. Water temperature ranged from 2.1–9.5°C. Note the decreased range of the Y-axis relative to Fig 3A. For clarity, points are jittered about the day of sampling. Values for day 1 all were zero and are not displayed. Polystyrene (PS) was added to the list of substrates referenced in Fig 3A.

In both experiments, biofilm was visible on submerged plastic by day four. In Colonization Experiment #1, concentrations of putative *Vibrio* spp. on the four plastic substrates and glass increased from 0 CFUs/cm^2^ on day 1 to between 500 and 2,000 CFUs/cm^2^ by day 16 and remained approximately the same or were slightly lower on day 30 (Fig 3A). Two-way ANOVA showed that concentrations on the five substrates (4 plastics and glass) did not differ significantly among themselves, but that there was a significant effect of time (days 2, 4, 16, 30; S2 Table). Mean concentrations of *Vibrio* spp. in paired water samples ranged between 3 CFUs/ml (day 16) and 250 CFUs/ml (day 1). On days 16 and 30, *Vibrio* concentrations were 500 to 1000 times greater on all substrates than in the water (Fig 3A).

In Colonization Experiment #2, when water temperatures were lower, concentrations of putative *Vibrio* spp. on the various substrates increased to a lesser extent, from 0 CFUs/cm^2^ on day 1 to between 7 and 24 CFUs/cm^2^ by day 31 (Fig 3B). There was a significant effect of time (days 2, 4, 9, 17, 31), concentrations on PS sometimes were lower than on the other five substrates, and the interaction term was significant (S3 Table). Mean concentrations of *Vibrio* spp. in paired water samples ranged between 1 CFUs/ml (day 16) and 13 CFUs/ml (day 1) and were greater than those on all substrates until day 31, when they were roughly equal for substrates and water, except for PS, which was consistently lower or lowest (Fig 3B).

### Comparison of CHROMagar identification and PCR results

Taken together, environmental samples and colonization experiments yielded a total of 384 putative *Vibrio* spp. isolates based on growth on CHROMagar^TM^. Of the amplifiable DNA samples from these isolates, 263 were PCR-confirmed as *Vibrio* spp. Of the PCR-confirmed *Vibrio* spp., 97 were further distinguished as *V*. *cholerae* (n = 5), *V*. *vulnificus* (n = 25), or *V*. *parahaemolyticus* (n = 67), indicating a low correlation (37%) between chromogenic identification of CFUs on CHROMagar^TM^ and *Vibrio* species identification using PCR. PCR-confirmed *V*. *vulnificus* and *V*. *parahaemolyticus* were found in water and on all substrates. *V*. *cholerae* was found in water samples, but on substrates, only on HDPE and PP. Given its small number of isolates (n = 5), however, we cannot speculate as to substrate specificity in this species.

### Antibiotic resistance testing

Antibiotic-resistance profiles for six antibiotics were determined for 76 of the 97 PCR-confirmed isolates. Profiles were incomplete for the remaining 21, for reasons of non-criterion growth. The most common forms of resistance were to ampicillin and rifampin (Table 2). Isolates were principally susceptible to tetracycline, gentamicin, and chloramphenicol. *V*. *parahaemolyticus* and *V*. *cholerae* isolates showed the highest resistance, while *V*. *vulnificus* isolates were more susceptible overall. For example, 89% (48 isolates) of *V*. *parahaemolyticus*, and 67% (2 isolates) of *V*. *cholerae* isolates showed resistance to ampicillin, whereas only 11% (2 isolates) of *V*. *vulnificus* isolates were resistant (Table 2). Nearly one third of isolates were resistant to multiple antibiotics (n = 25; 33%).

**Table 2:** *Vibrio* spp. isolates (n = 76) resistant, intermediate, or susceptible to ampicillin (AM), streptomycin (S), rifampin (RA), tetracycline (TE), gentamicin (GM), and chloramphenicol (C). Values are shown for number of isolates and corresponding percentage (in parentheses). Bolding indicates the most common forms of resistance. A) *V*. *parahaemolyticus*, *B) V*. *cholerae*, *C) V*. *vulnificus*.

A. *V*. *parahaemolyticus* (n = 54)						
	AM	GM	S	RA	C	TE
Resistant	**48 (89)**	2 (4)	3 (6)	**20 (37)**	0 (0)	0 (0)
Intermediate	4 (7)	10 (19)	33 (61)	11 (20)	2 (4)	4 (7)
Susceptible	2 (4)	42 (78)	18 (33)	23 (43)	52 (96)	50 (93)
B. *V*. *cholerae* (n = 3)						
	AM	GM	S	RA	C	TE
Resistant	**2 (67)**	0 (0)	**2 (67)**	**2 (67)**	0 (0)	0 (0)
Intermediate	1 (33)	2 (67)	1 (33)	1 (33)	1 (33)	0 (0)
Susceptible	0 (0)	1 (33)	0 (0)	0 (0)	2 (67)	3 (100)
C. *V*. *vulnificus* (n = 19)						
	AM	GM	S	RA	C	TE
Resistant	2 (11)	1 (5)	2 (11)	4 (21)	0 (0)	1 (5)
Intermediate	0 (0)	3 (16)	6 (32)	6 (32)	2 (11)	0 (0)
Susceptible	17 (89)	15 (79)	11 (58)	9 (47)	17 (89)	18 (95)

**Fig 4:**
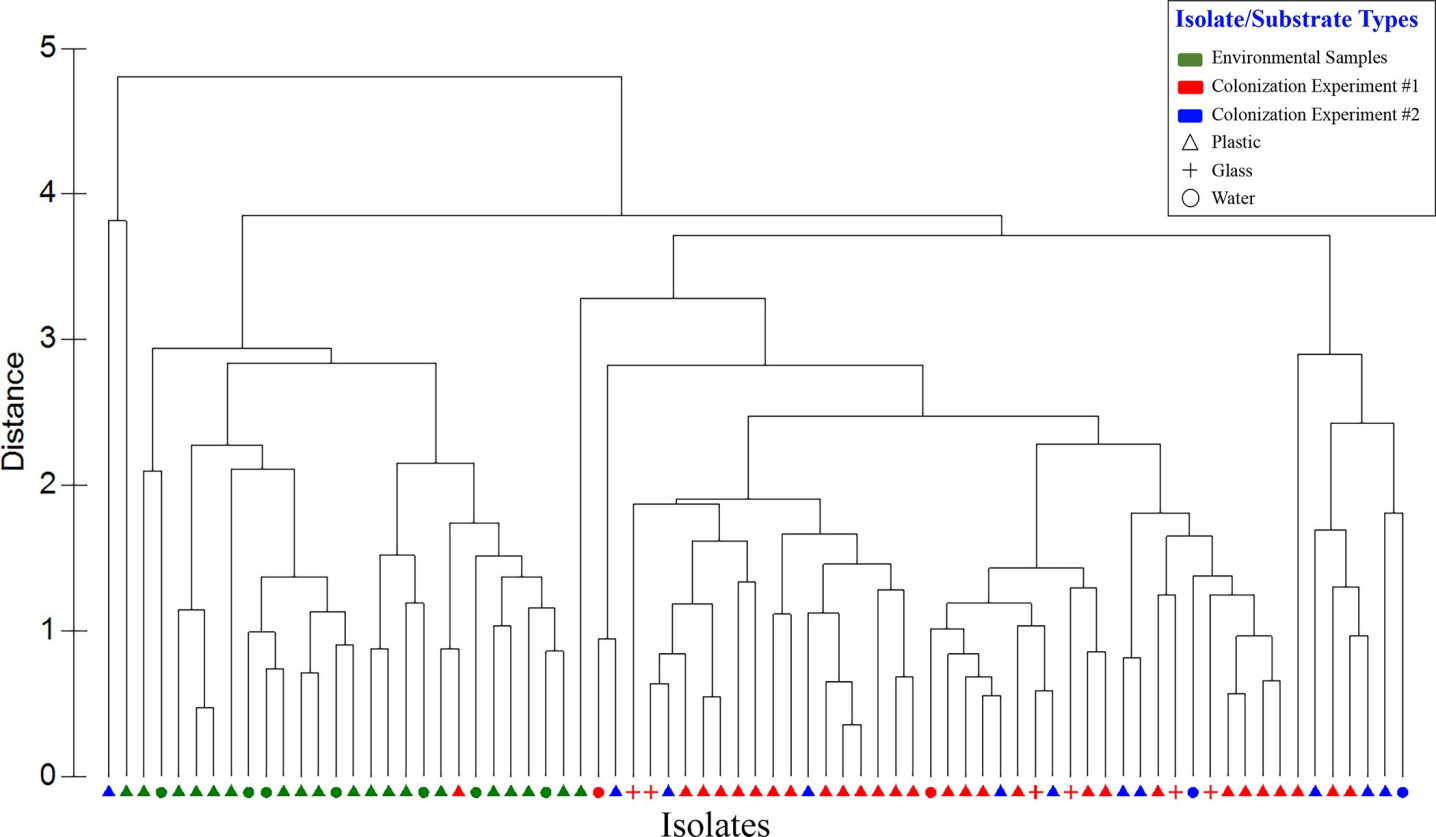
Hierarchical clustering dendrogram showing isolates’ relationship based on their antibiotic susceptibility profiles. ZOI data for each isolate was compared with that of all other isolates using Euclidean distance similarity, then clustered using a group average algorithm. Red symbols, Colonization Experiment #1; blue symbols, Colonization Experiment #2; green symbols, environmental samples. Plastic substrates (all types), triangles; glass substrate; +; water, filled circles.

**Fig 5:**
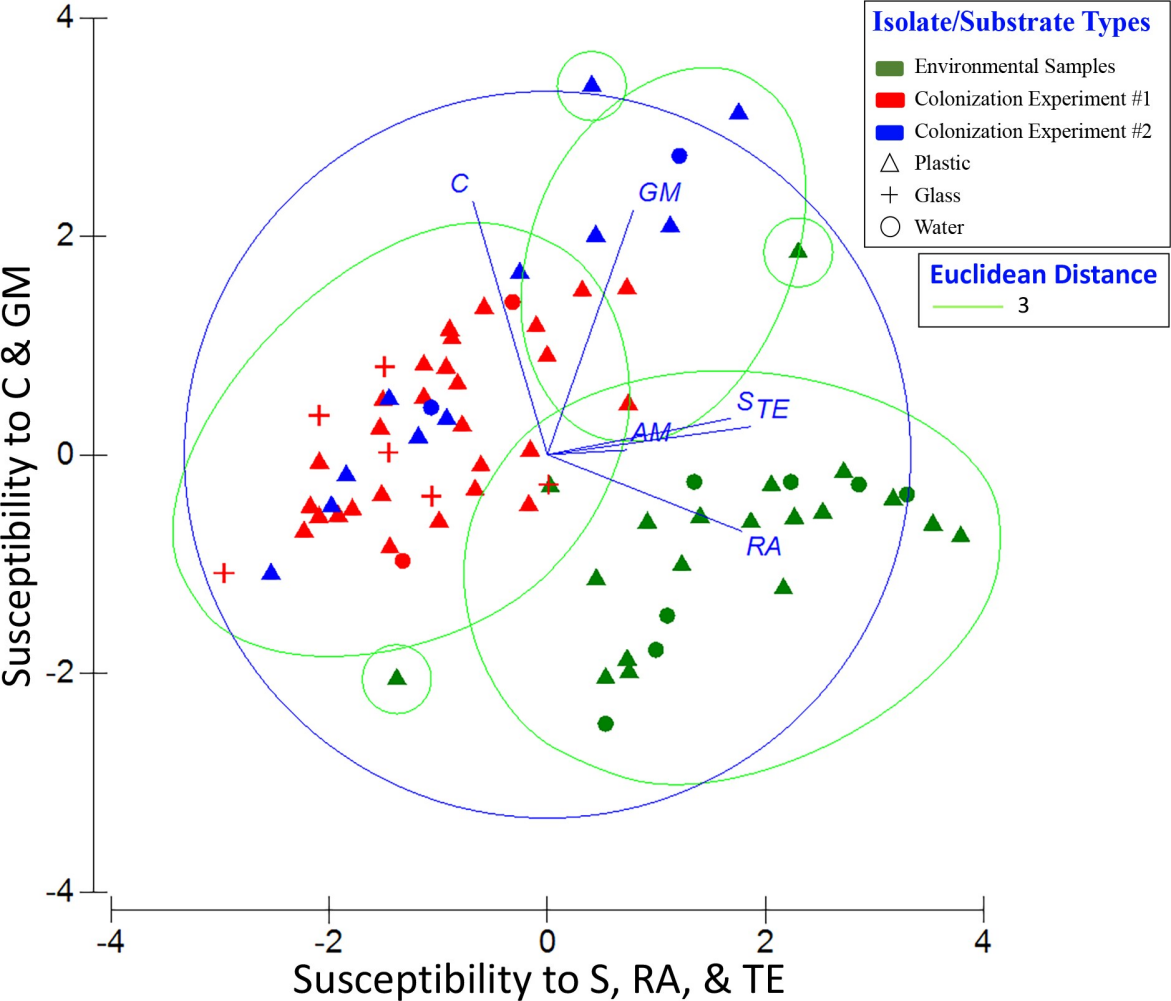
Principal components analysis (PCA) of antibiotic profiles of *Vibrio* spp. (n = 97) by sample type. PC1 represents increasing susceptibility to streptomycin (S), rifampin (RA), and tetracycline (TE), and PC2 represents increasing susceptibility to chloramphenicol (C) and gentamicin (GM). Eigenvectors for each antibiotic are shown as lines adjacent to the corresponding labels. Symbols as in Fig 4. The PCA is overlain with Euclidean distance (value of 3) from the cluster analysis (Fig 4).

**Fig 6:**
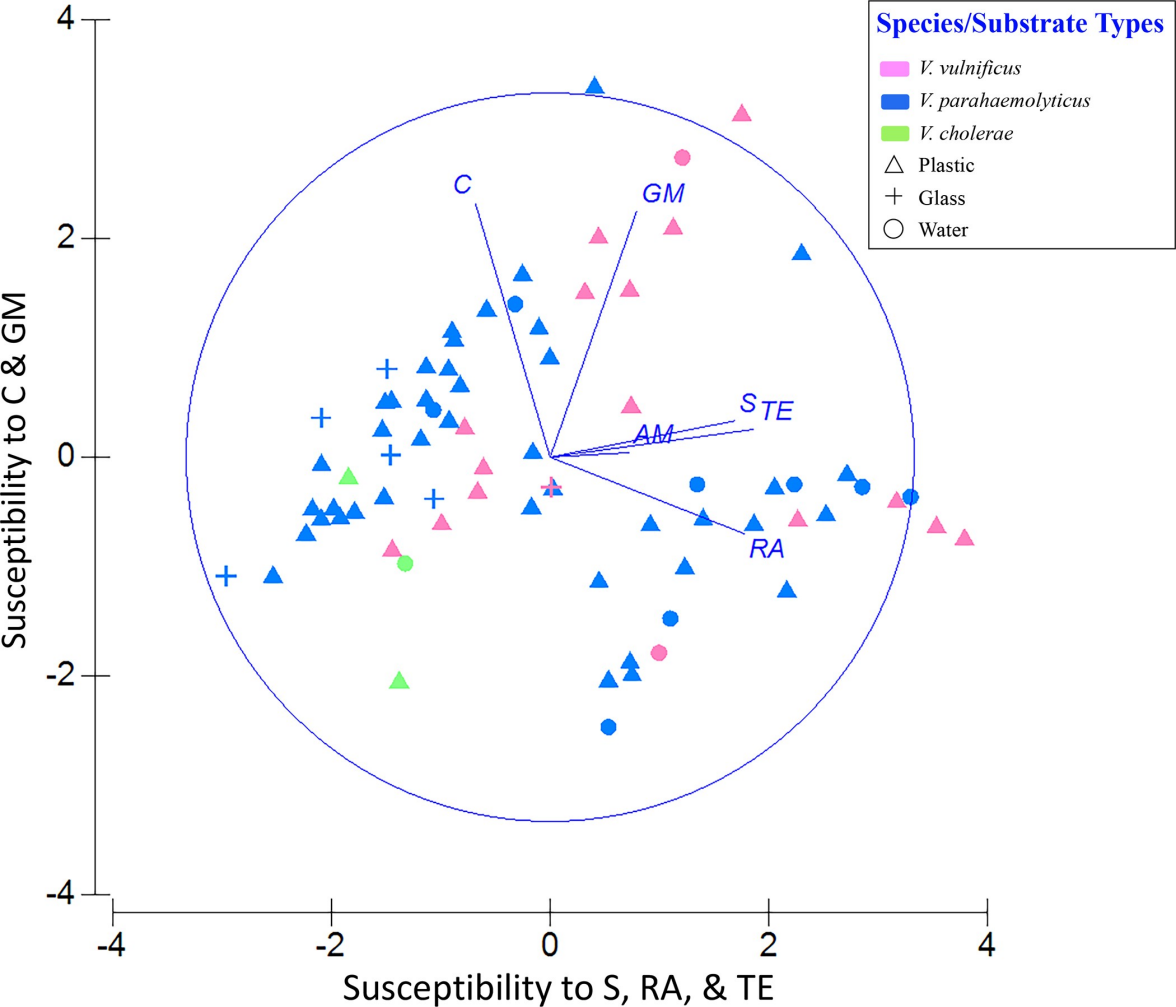
Principal components analysis (PCA) of antibiotic profiles identifying the 97 *Vibrio* isolates. PC1 and PC2 as in Fig 5. Eigenvectors for each antibiotic are shown as lines. Isolates are color coded: magenta, *V*. *vulnificus*; blue, *V*. *parahaemolyticus*; green, *V*. *cholerae*.

In both a hierarchical cluster analysis (Fig 4) and a 2-D PCA plot in which resistance profiles were categorized by sample type (Fig 5), resistance patterns of the 76 isolates emerged as two distinct groups—one from colonization experiments and the other from environmental samples. Within these groups, there was no apparent pattern with respect to sampling date and no distinction between isolates from water versus plastics. (More detailed versions of Figs 4 and 5, ones showing sample numbers, are presented in S1 and S2 Figs). Principal component (PC) 1 explained 44.9% of the variance and was influenced most by tetracycline, rifampin, and streptomycin, with loading values of 0.92, 0.88, and 0.83, respectively (S4 Table). PC2 explained 22.9% of the variance and was more influenced by chloramphenicol and gentamicin. Combining PCs 1, 2, and 3 explained 83.8% of the variance in the data set. A second PCA plot identifying the *Vibrio* spp. associated with each point showed those isolates exhibiting the greatest susceptibility to tetracycline, rifampin, and streptomycin had high, positive scores along PC1 (Fig 6). Isolates exhibiting greatest susceptibility to chloramphenicol and gentamicin had high, positive scores along PC2. (A more detailed version of Fig 6 is presented in S3 Fig). Wilcoxon rank-sum tests showed that isolates from Colonization Experiment #1 were more resistant to streptomycin and tetracycline than were isolates from environmental samples, while environmental samples were more resistant to gentamicin and chloramphenicol (S5 Table). In Colonization Experiment #2, isolates were more resistant, compared to those from environmental samples, only for streptomycin (S5 Table).

### Biofilm DNA extractions and sequencing

Nanodrop concentrations of sequenced DNA ranged between 0.6 and 45.1 ng/μl. Concentrations were low in the early stages (days 2 and 4) and increased over time. Bacterial colonization was detected with DNA sequencing as early as day 2. Approximately 1 million Illumina unpaired sequence reads were obtained for all 136 samples. After quality processing and normalization, 826,110 reads remained, with 243,271 unique sequences at ≥97% similarity. Of the 136 samples, 7 samples were sequenced for quality control and therefore excluded in further analyses.

### Sample coverage and diversity

Estimates of Good’s coverage ranged from 97.6% (1.2.LDPE1) to 99.8% (2.2.LDPE1) (S6 Table), demonstrating sufficient sampling coverage. The number of unique OTUs ranged from 56 (1.2.Glass1) to 326 (2.17.PC2) and increased over time in both colonization experiments (S6 Table). Environmental samples displayed no temporal pattern for bacterial richness (S6 Table). At the close of Colonization Experiment #1 (day 30), 1-way ANOVA showed no significant difference in the number of unique OTUs among the 4 plastics and glass (df = 4,10; F = 2.144; p = 0.150). Similarly, in Experiment #2, on 31 days there was no difference among 5 plastics and glass, and all biofilm communities had more unique OTUs than those in water samples (ANOVA, df = 6,14; F = 6.218; p = 0.002). The Shannon diversity index ranged from 1.19 (2.4.PS2) to 3.74 (2.2.PP2) across all samples (S6 Table). There was no difference in the index at the close of Experiment 1 among the 4 plastics and glass (ANOVA, df = 4,10; F = 0.909; p = 0.495). In Experiment 2, however, the ANOVA comparing diversity in bacterial communities of 5 plastics, glass, and water was significant (df = 6,14; F = 4.646; p = 0.008) and Tukey’s HSD test indicated the index was lower on glass than on LDPE or PC (both comparisons p<0.05) and water (p<0.01).

### Relative abundances of dominant taxa

**Fig 7:**
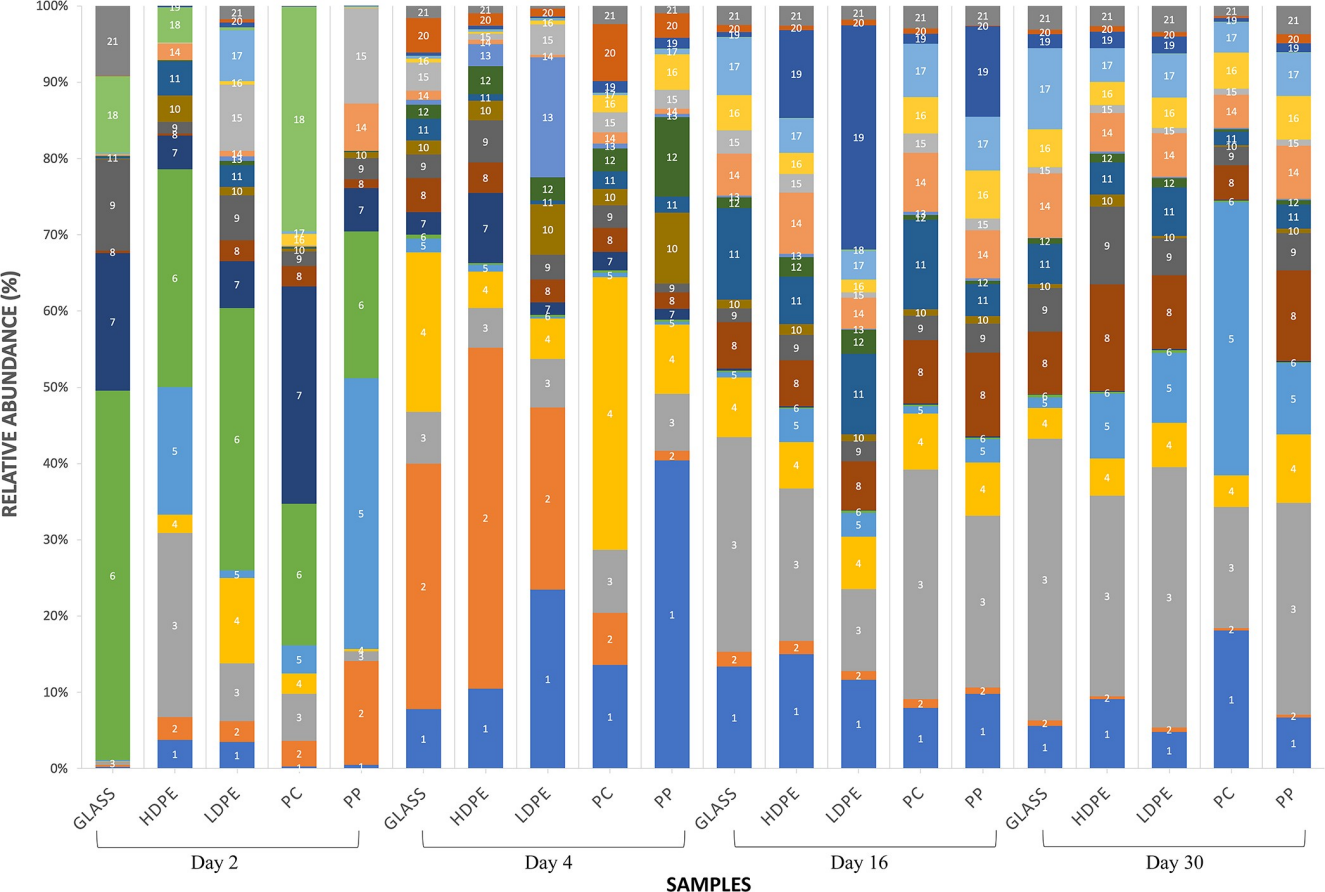
Relative abundances of sequenced Bacteria in Colonization Experiment #1. Substrate types: low-density polyethylene (LDPE), high-density polyethylene (HDPE), polypropylene (PP), polycarbonate (PC), and glass (G). Paired water samples were not collected. The most abundant OTUs are listed as follows: (1) Bacteria, Bacteria Unclassified; (2) Bacteria, Proteobacteria, Gammaproteobacteria, Pseudomonadales, Pseudomonadaceae, *Pseudomonas;* (3) Bacteria, Proteobacteria, Gammaproteobacteria, Oceanospirillales, Oceanospirillaceae; (4) Bacteria, Bacteroidetes, Flavobacteria, Flavobacteriales, Flavobacteriaceae, Tenacibaculum; (5) Bacteria, Proteobacteria, Gammaproteobacteria, Pseudomonadales, Pseudomonadaceae, *Pseudomonas;* (6) Bacteria, Bacteroidetes, Flavobacteria, Flavobacteriales, Flavobacteriaceae, *Maribacter;* (7) Bacteria, Verrucomicrobia, Verrucomicrobiae, Verrucomicrobiales, Rubritaleaceae, *Rubritalea;* (8) Bacteria, Bacteroidetes, Flavobacteria, Flavobacteriales, Flavobacteriaceae, *Flavobacterium;* (9) Bacteria, Proteobacteria, Gammaproteobacteria, Alteromonadales, Pseudoalteromonadaceae, *Pseudoalteromonas;* (10) Bacteria, Proteobacteria, Gammaproteobacteria, Oceanospirillales, Oceanospirillaceae, *Oleibacter;* (11) Bacteria, Actinobacteria, Actinobacteria, Actinomycetales, Corynebacteriaceae, *Corynebacterium;* (12) Bacteria, Proteobacteria, Alphaproteobacteria, Rhodobacterales, Rhodobacteraceae; (13) Bacteria, Bacteroidetes, Flavobacteria, Flavobacteriales, Flavobacteriaceae; (14) Bacteria, Proteobacteria, Gammaproteobacteria, Alteromonadales, Alteromonadaceae, *Glaciecola;* (15) Bacteria, Bacteria Unclassified; (16) Bacteria, Proteobacteria, Gammaproteobacteria, Vibrionales, Vibrionaceae, *Vibrio;* (17) Bacteria, Proteobacteria, Alphaproteobacteria, Sphingomonadales, Sphingomonadaceae, *Sphingomonas;* (18) Bacteria, Bacteroidetes, Flavobacteria, Flavobacteriales, Flavobacteriaceae, *Polaribacter;* (19) Bacteria, Proteobacteria, Alphaproteobacteria, Sphingomonadales, Erythrobacteraceae, *Erythrobacter;* (20) Bacteria, Proteobacteria, Alphaproteobacteria, Rhodobacterales, Rhodobacteraceae; (21) Bacteria, Proteobacteria, Alphaproteobacteria, Rhodobacterales, Rhodobacteraceae.

**Fig 8:**
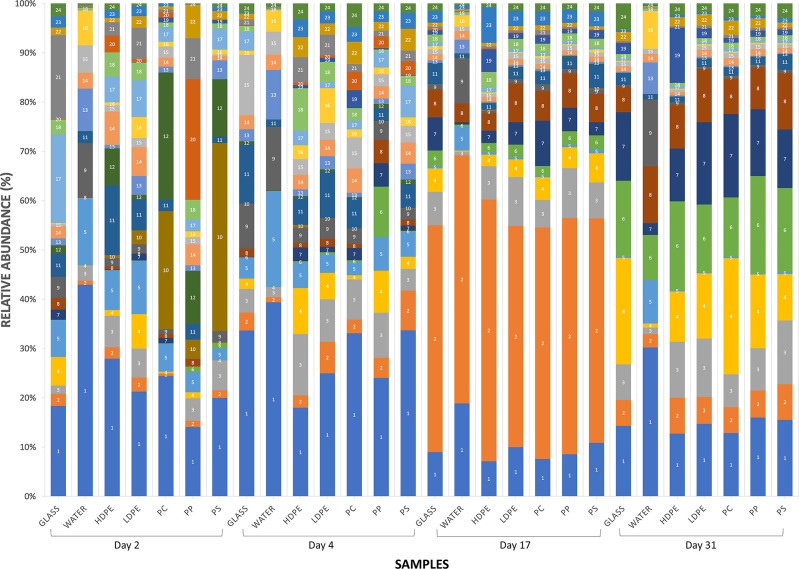
Relative abundances of sequenced Bacteria in Colonization Experiment #2. As in Fig 7, with the addition of polystyrene (PS) as a substrate and paired water samples. The most abundant OTUs are listed as follows: (1) Bacteria, Proteobacteria, Gammaproteobacteria, Oceanospirillales, Oceanospirillaceae; (2) Bacteria, Bacteria Unclassified; (3) Bacteria, Proteobacteria, Alphaproteobacteria, Sphingomonadales, Sphingomonadaceae, Sphingomonas; (4) Bacteria, Bacteria Unclassified; (5) Bacteria,Verrucomicrobia, Verrucomicrobiae, Verrucomicrobiales, Verrucomicrobiaceae, Luteolibacter; (6) Bacteria, Bacteroidetes, Flavobacteria, Flavobacteriales, Flavobacteriaceae, Flavobacterium, (7) Bacteria, Bacteroidetes, Flavobacteria, Flavobacteriales, Flavobacteriaceae, (8) Bacteria, Proteobacteria, Betaproteobacteria, Burkholderiales, Comamonadaceae, Delflia, (9) Bacteria, Proteobacteria, Alphaproteobacteria, Rhodobacterales, Rhodobacteraceae, Loktanella; (10) Bacteria, Proteobacteria, Gammaproteobacteria, Oceanospirillales, Oceanospirillaceae; (11) Bacteria, Bacteroidetes, Flavobacteria, Flavobacteriales, Flavobacteriaceae, Tenacibaculum, (12) Bacteria, Bacteroidetes, Flavobacteria, Flavobacteriales, Flavobacteriaceae, Polaribacter; (13) Bacteria, Proteobacteria, Betaproteobacteria, Burkholderiales, Comamonadaceae; (14) Bacteria, Bacteroidetes, Flavobacteria, Flavobacteriales, Flavobacteriaceae, Flavobacterium; (15) Bacteria, Proteobacteria, Gammaproteobacteria, Alteromonadales, Pseudoalteromonadaceae, Pseudoalteromonas, (16) Bacteria, Proteobacteria, Gammaproteobacteria, Pseudomonadales, Pseudomonadaceae, Pseudomonas; (17) Bacteria, Bacteroidetes, Flavobacteria, Flavobacteriales, Flavobacteriaceae, Maribacter; (18) Bacteria, Bacteria Unclassified; 19) Bacteria, Proteobacteria, Alphaproteobacteria, Rhodobacterales, Rhodobacteraceae; (20) Bacteria, Proteobacteria, Gammaproteobacteria, Vibrionales, Vibrionaceae, Vibrio; (21) Bacteria, Proteobacteria, Gammaproteobacteria, Alteromonadales, Alteromonadaceae, Glaciecola; (22) Bacteria, Actinobacteria, Actinobacteria, Actinomycetales, Corynebacteriaceae, Corynebacterium, (23) Bacteria, Bacteroidetes, Flavobacteria, Flavobacteriales, Flavobacteriaceae, Maribacter; (24) Bacteria, Proteobacteria, Alphaproteobacteria, Rhodobacterales, Rhodobacteraceae.

**Fig 9:**
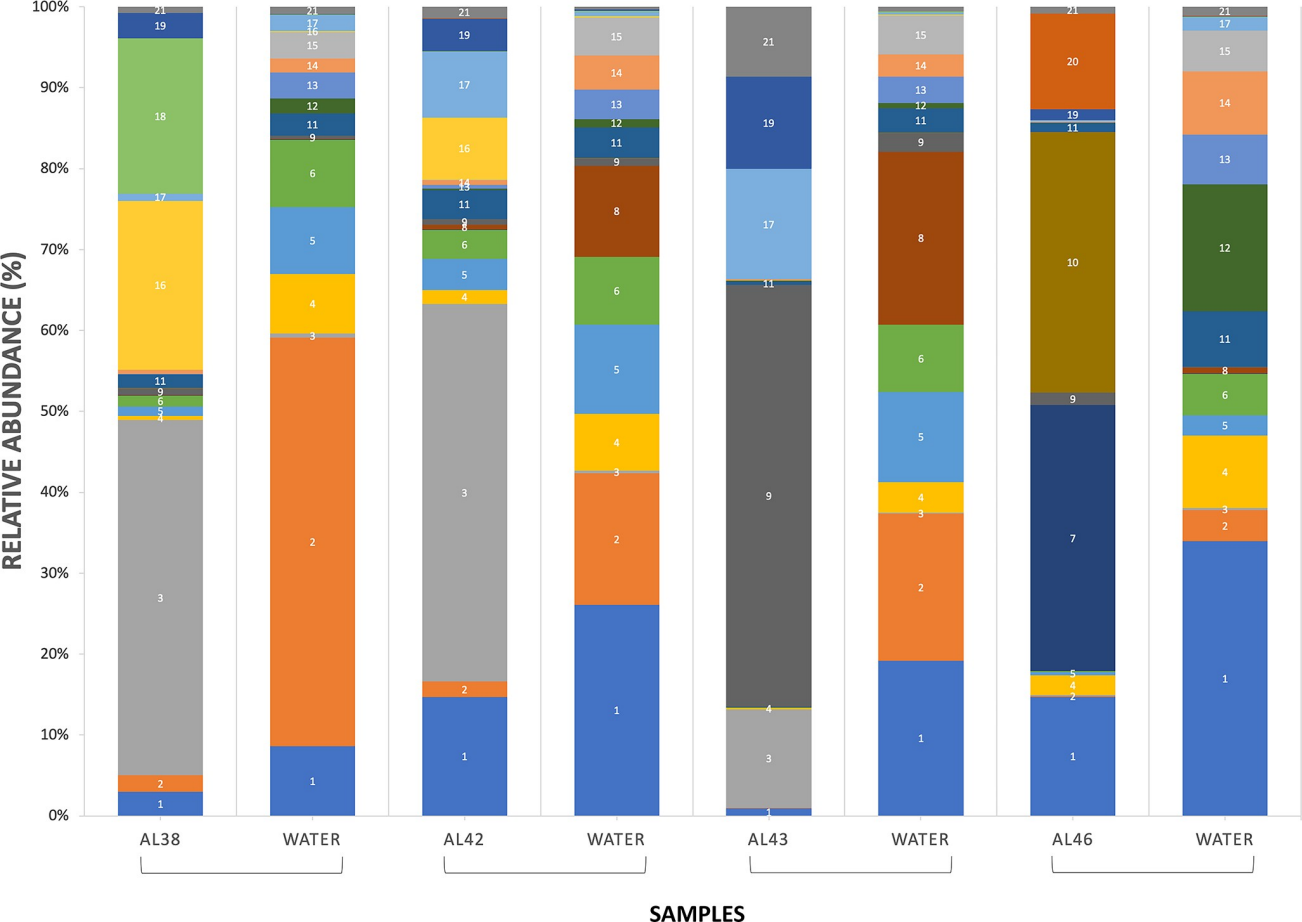
Relative abundances of sequenced Bacteria on polyethylene microplastics (ATR-FTIR confirmed) and paired water samples. Sample number is indicated as AL##. The most abundant OTUs are listed as follows: (1) Bacteria, Proteobacteria, Gammaproteobacteria, Oceanospirillales, Oceanospirillaceae; (2) Bacteria, Proteobacteria, Gammaproteobacteria, Alteromonadales, Alteromonadaceae, *Glaciecola;* (3) Bacteria, Proteobacteria, Gammaproteobacteria, Pseudomonadales, Pseudomonadaceae, *Pseudomonas;* (4) Bacteria, Proteobacteria, Gammaproteobacteria, Alteromonadales, Pseudoalteromonadaceae, *Pseudoalteromonas;* (5) Bacteria, Bacteroidetes, Flavobacteria, Flavobacteriales, Cryomorphaceae, *Lishizhenia;* (6) Bacteria, Proteobacteria, Gammaproteobacteria, Alteromonadales, Alteromonadaceae, *Glaciecola;* (7) Bacteria, Proteobacteria, Alphaproteobacteria, Rhodobacterales, Rhodobacteraceae, *Pseudoruegeria;* (8) Bacteria, Proteobacteria, Gammaproteobacteria, Vibrionales, Vibrionaceae, *Vibrio;* (9) Bacteria, Bacteroidetes, Flavobacteria, Flavobacteriales, Flavobacteriaceae, *Maribacter;* (10) Bacteria, Verrucomicrobia, Verrucomicrobiae, Verrucomicrobiales, Rubritaleaceae, *Rubritalea;* (11) Bacteria, Bacteroidetes, Flavobacteria, Flavobacteriales, Flavobacteriaceae, *Flavobacterium;* (12) Bacteria, Proteobacteria, Gammaproteobacteria, Pseudomonadales, Pseudomonadaceae, *Pseudomonas; (13)* Bacteria, Proteobacteria, Betaproteobacteria, Betaproteobacteria Unclassified; (14) Bacteria, Proteobacteria, Betaproteobacteria, Burkholderiales, Comamonadaceae; (15) Bacteria, Verrucomicrobia, Verrucomicrobiae, Verrucomicrobiales, Verrucomicrobiaceae, *Luteolibacter;* (16) Bacteria, Proteobacteria, Alphaproteobacteria, Rhodobacterales, Rhodobacteraceae; (17) Bacteria, Bacteroidetes, Flavobacteria, Flavobacteriales, Flavobacteriaceae, *Tenacibaculum;* (18) Bacteria, Bacteroidetes, Flavobacteria, Flavobacteriales, Flavobacteriaceae, *Krokinobacter;* (19) Bacteria, Bacteria Unclassified; (20) Bacteria, Actinobacteria, Actinobacteria, Acidimicrobiales, Acidimicrobiaceae, *Ilumatobacter;* (21) Bacteria, Proteobacteria, Alphaproteobacteria, Rhodobacterales, Rhodobacteraceae.

Overall, fourteen bacterial classes were found in water and biofilms and 171 genera were identified. Among all sequence reads, 12% at the class level and 18% at the family level could not be classified. Relative abundances were heavily weighted towards Gram-negative organisms. Across all samples, *Gammaproteobacteria* were the largest constituent of sequences (30%), followed by *Bacteroidetes* (28%) and *Alphaproteobacteria* (20%). This overall pattern generally manifested in Colonization Experiment #1 (Fig 7), Colonization Experiment #2 (Fig 8), and select environmental samples, specifically, paired samples of PE microplastics (ATR-FTIR confirmed) and estuarine water (Fig 9). The greatest deviation from this pattern occurred on day 17 of Colonization Experiment #2, when unclassified bacteria increased greatly in relative abundance. Unclassified bacteria were the next most dominant group in Colonization Experiment #1 (13% overall; Fig 7) and #2 (15% overall; Fig 8), followed by *Actinobacteria* (5%) and *Verrucomicrobia* (4%) (Experiment #1), and *Betaproteobacteria* (6%) and *Verrucomicrobia* (4%) (Experiment #2). For the paired PE microplastics and water samples, the next most dominant bacterial classes were *Betaproteobacteria* (6%), *Verrucomicrobia* (5%), and *Actinobacteria* (3%) (Fig 9). Compared to bacteria on PE, the water communities always exhibited greater and lesser dominance, respectively, of *Gammaproteobacteria* and *Alphaproteobacteria*.

Members of the *Verrucomicrobia* phylum were identified in communities from plastics and water over the course of both colonization experiments. They were present in much higher relative abundances, however in the early stages of biofilm formation (days 2 and 4) before diminishing in plastic-associated communities, especially in Colonization Experiment #1. The biofilm of a PE microplastic sample, AL46, contained a high proportion of *Verrucomicrobia* (20%) (Fig 9).

Within *Gammaproteobacteria*, the most commonly retrieved bacterial orders overall were *Oceanospirillales* (43%), *Alteromonadales* (22%), and *Pseudomonadales* (21%). *Vibrionales*, the order containing *Vibrio* spp., represented 7% of the total *Gammaproteobacteria* sequences found. The greatest relative abundances of *Vibrio* spp. were most commonly found on PP, with *Vibrio* constituting almost 25% of the PP sample on day 2 (Fig 8). Of all sequence reads, *Oceanospirillales* accounted for 15%, *Alteromonadales*, 5%, *Pseudomonadales*, 7%, and *Vibrionales*, 2%. Among *Bacteroidetes*, *Flavobacteria* dominated the sequences found (24% of total) and within *Alphaproteobacteria*, *Rhodobacterales* constituted 15% of all sequence reads.

**Fig 10:**
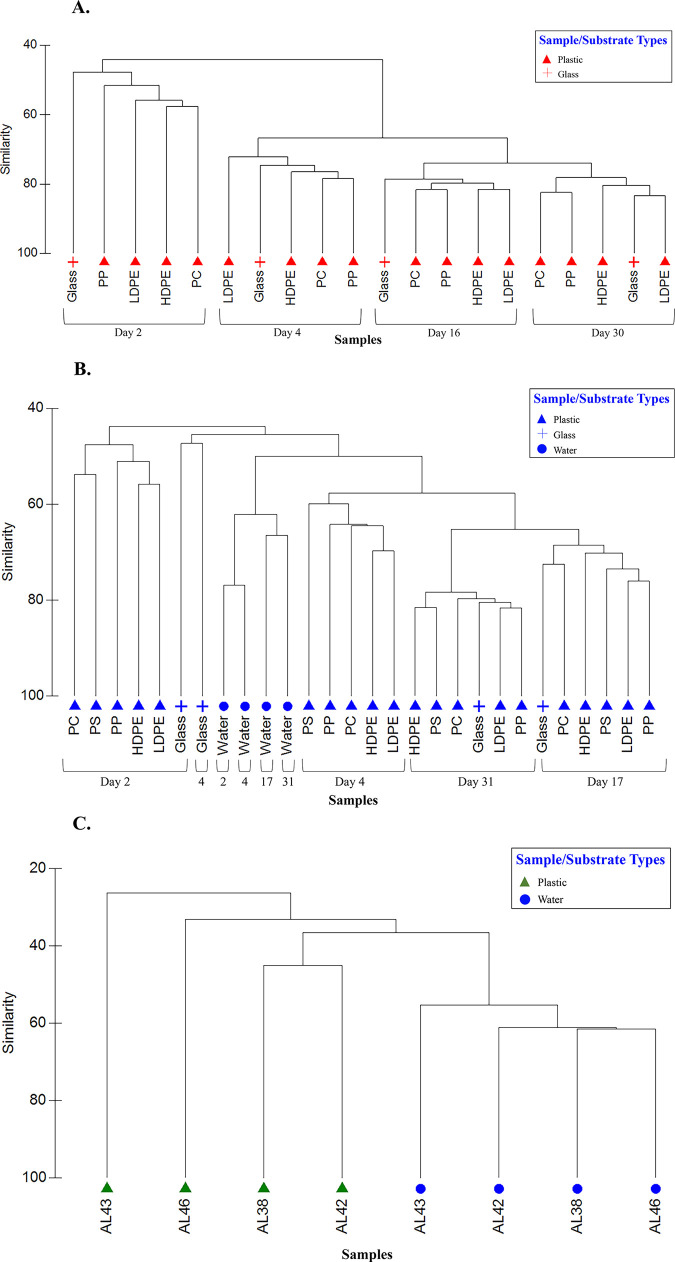
Hierarchical clustering dendrogram of bacterial communities showing sample relationship based on sequence data. Abundance data (Log (x+1)) was compared between samples using Bray-Curtis similarity, then clustered using a group-average algorithm. A) Colonization Experiment #1; B) Colonization Experiment #2; C) environmental samples (polyethylene microplastics and paired water samples). Substrate types: low-density polyethylene (LDPE), high-density polyethylene (HDPE), polypropylene (PP), polycarbonate (PC), glass (Glass), and polystyrene (PS).

Colonizing Bacteria showed very strong affinity by sampling date rather than substrate with the exception of communities on glass (days 2 and 4 only) in Colonization Experiment #2 (Fig 10A and 10B). There was no indication of plastic-specific communities in either colonization experiment. Further, communities on plastics were not consistently different from those on glass. In Colonization Experiment #2, all water samples formed a cluster most similar to biofilms from glass and plastic substrates from days 4, 17, and 31. Communities on PE microplastics exhibited high fidelity to one another and were distinct from communities in paired water samples (Fig 10C).

## Discussion

### Microplastics collected from the estuarine environment

In this study, microplastics were collected principally to facilitate subsequent study of their attached Bacteria. In net tows from a dock, we collected 51 pieces of putative microplastics, an average of 0.07 pieces/m^3^. In rivers of the upper Chesapeake Bay, concentrations of microplastics ranged over 3 orders of magnitude (<1.0 to >560 g/km^2^), were positively correlated with population density, and occurred in greatest concentrations at three of four sites shortly after major rains [34]. Along a broad swath of the Chesapeake Bay mainstem, Bikker et al. (2020) [35] also reported a wide range of microplastic concentrations, from 0.007 to 1.245 particles/m^3^. In general, these values are lower to much lower than those reported for the European coast and the North Atlantic Subtropical Gyre (between 13 to 501 items/m^3^) [36], although approximately 40% of those microplastic counts were fibers, a form not considered in our analyses.

Of those microplastic plastics identified by ATR-FTIR, PE was most commonly recovered (13 of 23 pieces; Table 1). Multiple other studies report PE’s dominance of microplastics in aquatic environments, likely due in part to its extremely buoyant characteristics, as with PP and PS [14, 17, 37, 38]. Furthermore, a great amount of PE is available; it and PP are the most produced plastics across the globe, primarily for single-use packaging, with polyethylene terephthalate (PET) and PS closely following [38].

ATR-FTIR identification of the polymeric composition of microplastics was not possible in 6 of 23 cases, because strongly attached biofilms, apparently resistant to treatment with an oxidant, interfered with the analysis. Others have reported such analytical issues with biofilms on plastics collected from the environment [39].

### Microbial communities on plastic pollution

Zettler et al. (2013) [14] first used next-generation sequencing (NGS) to examine microbial communities attached to marine plastic. Since then, others have employed NGS to examine diversity and structure of microbial communities on different plastics in the environment [15, 17, 40, 41]. An emergent concept is that the microbial communities on marine plastics differ significantly from those in surrounding seawater [15, 42]. Results here reinforce that concept (Figs 9, 10B and 10C), consistent with a paradigm in microbial ecology established for natural organic particles in aquatic environments, e.g., Lyons et al. (2010) [43].

In the present study, diverse bacterial communities colonizing five plastic types were much more distinct across time than they were among one another (Fig 10A and 10B). There was no suggestion of plastic-specific assemblages. Furthermore, plastic-associated communities were not standardly distinguishable from glass-attached communities. The lack of consistent, significant differences among biofilms on different plastic substrates was also reported by Oberbeckmann et al. (2016) [42], who posited drivers of biofilm community composition are principally the availability of a surface and the environmental conditions present at the time of colonization, rather than the type of plastic polymer [42]. Indeed, Oberbeckmann et al. (2014) [39] and Amaral-Zettler et al. (2015) [16] considered that microbial community composition on plastics varies more with geographical location and season than by plastic type. Recently, Oberbeckmann et al. (2018) demonstrated convincingly that the degree of specificity in substrate colonization depends on ambient environmental conditions [41].

In both colonization experiments, bacterial communities were similar across substrates following approximately 2.5 weeks and 1 month of immersion (Fig 10A and 10B). This similarity suggests the communities’ composition not only converged over time (no evidence of plastic-specific bacterial assemblages), but became more stable. In addition, we note that *Verrucomicrobia* had greater relative abundances in the early days of the colonization experiments, then greatly decreased. We conjecture that microplastics with higher concentrations of this taxa may indicate recently introduced plastic pollution. If so, then microplastic piece AL46 (Fig 9) would be predicted to be “younger” (with respect to time in the water) than the other three PE pieces pictured, given the greater proportion of *Verrucomicrobia* in its attached biofilm. In addition to time in the environment, however, many other factors such as plastic additives, bio-accumulated persistent organic pollutants, biofilm formation stages, and ingestion by animals may play roles in the variation seen among microbial colonization of plastic pollution [16]. Determining how and the degree to which each of these factors contributes to colonization of marine plastic represents future research questions.

### Plastic pollution and *Vibrio* spp.

As expected in a temperate-climate estuary, *Vibrio* spp. were found in all water samples and on every substrate examined, plastics and glass. Furthermore, three potentially pathogenic species, *V*. *cholerae*, *V*. *vulnificus*, and *V*. *parahaemolyticus*, were cultured for the first time from biofilm communities on estuarine plastics. In two colonization experiments, *Vibrio* concentrations on all substrates increased over time, although concentrations were lower in the experiment that began in January, when water temperatures were colder (Fig 3A and 3B). Vibrios thrive in warm coastal waters and their concentrations are known to be positively correlated with water temperature [44]. In several instances, *Vibrio* spp. enumerated from substrates exceeded their concentrations in water by two orders of magnitude (Figs 2 and 3A). Because biofilm formation is known to provide survival advantages to aquatic microorganisms [45], it is not surprising to find enriched concentrations on plastic substrates.

Kirstein et al. (2016) confirmed the presence of *Vibrio* spp. on 13% of the marine microplastics they collected [17]. They detected *V*. *parahaemolyticus* on 12 microplastics, and in contrast to the present study, observed *V*. *vulnificus* and *V*. *cholerae* only in water samples. In the present study’s NGS results, the greatest relative abundances of *Vibrio* spp. occurred on PP and in water (Figs 8 and 9). Zettler et al. (2013) identified a member of the genus *Vibrio* constituting nearly 24% of one PP sample [14]. Taken together, these studies confirm the ubiquity of vibrios on marine and estuarine microplastics and suggest that PP may be a favored substrate. This result may be due to the plastic’s structure, surface charge, manufacturing protocol, lability, or some combination of variables.

### Antibiotic resistance of *Vibrio* spp.

The potential for plastic in aqueous environments to serve as a vector for pathogenic organisms is compounded by the possibility for its dissemination of antibiotic-resistance genes. The transport and transfer of antibiotic resistance on marine plastic has received little attention but is considered an urgent topic to address [41, 46]. Arias-Andres et al. (2018) first reported horizontal transfer of antibiotic-resistance genes on marine plastic biofilms and determined that plasmid transfer is significantly greater on microplastics than in the surrounding water. Their study cautions about the potential for an exponential (100,000-fold) increase in the transfer of antibiotic resistance genes in aquatic environments [46].

In this study, we examined antibiotic resistance culturally, rather than through molecular methods. Overall, antibiotic-resistance profiles of *Vibrio* isolated from plastics in colonization experiments were no different than those from *Vibrio* isolated from the surrounding water column. There were, however, significant differences in profiles between isolates from colonization experiments and those from environmental samples, with more resistance overall seen in the former (Figs 5 and 6). Within these two groups, however, there was no discernible pattern with respect to sampling date and no clear distinction between isolates from water versus those from plastics. These differences may be influenced by location, therefore, as environmental samples were collected from the Elizabeth River and colonization experiments were conducted in the Lafayette River. Although the two sampling sites are only approximately 4 km apart and connected (one is a tributary of the other), the Virginia Zoological Park lies on the Lafayette River and its associated runoff is approximately 1500 meters upstream from the site used for colonization experiments. There may be more antibiotic-resistance genes inherent in this wastewater, since zoo animals are known to act as reservoirs of bacteria harboring antimicrobial resistance genes [47].

### Plastic pollution and other pathogens

Through analysis of NGS results, we identified other potential pathogens. Members of *Tenacibaculum*, a genus that harbors several fish pathogens [48], constituted 12% of the total *Bacteroidetes* discovered (Figs 7, 8 and 9). *Enterobacteriaceae*, a group that includes *Salmonella*, *Escherichia coli*, *Yersinia pestis*, *Klebsiella*, and *Shigella*, were present but constituted less than 1% of the total *Gammaproteobacteria* (Figs 7, 8 and 9). Of these genera, we detected only *Shigella*. Oberbeckmann et al. (2018) also found low (<0.5%) relative abundances of *Enterobacteriaceae* on plastic substrates compared to water [41]. And while not pathogens per se, concerns have been raised that other unfavorable organisms may be transported via plastics, including dinoflagellates that cause harmful algal blooms [11].

## Conclusions

In experiments that tracked bacterial colonization of plastics (LDPE, HDPE, PP, PC, and PS) in estuarine waters, we found no evidence of plastic-specific communities. Instead, time was the grouping factor for bacterial biofilms that developed on plastics. Colonizing communities as well as those on microplastics collected from the environment differed substantially from those in paired estuarine water samples, consistent with a paradigm in microbial ecology [43]. Significantly, we also demonstrated the presence of potential pathogens, especially three species of *Vibrio* bacteria (*V*. *vulnificus*, *V*. *parahaemolyticus*, and *V*. *cholerae)*, on plastics in the estuarine environment, supporting and expanding upon initial reports of vibrios on microplastics [14, 17]. Finally, our research is among the first to demonstrate antibiotic resistance of potential pathogens colonizing plastics in the estuarine environment, strengthening the contention that microplastics can serve as planktonic focal points of pathogens and horizontal gene transfer [17, 41, 46].

## Supporting information

S1 Table Temperature (°C) and salinity (ppt) values for A) Colonization Experiment #1, B) Colonization Experiment #2, and C) Environmental Samples.
(DOCX)

S2 Table ANOVA on concentration of putative *Vibrio* spp. in Colonization Experiment #1. Columns represent substrates (LDPE, HDPE, PP, PC, Glass) and rows represent days (2, 4, 16, 30).
(DOCX)

S3 Table ANOVA on concentration of putative *Vibrio* spp. in Colonization Experiment #2. Columns represent substrates (LDPE, HDPE, PP, PC, Glass, and PS) and rows represent days (2, 4, 9, 17, 31).
(DOCX)

S4 Table Loading values from principal component analysis of antibiotic susceptibility data for six antibiotics (abbreviated as in Table 2). The magnitude of loading, positive or negative, indicates the degree of influence the antibiotic has on each principal component. Loadings for the first three PCs are shown and for each, loadings having high absolute values are bolded.
(DOCX)

S5 Table Comparisons of Zones of Inhibition (ZOI) for six antibiotics (abbreviated as in Table 2) in isolates from colonization experiments versus isolates from environmental samples using a Wilcoxon rank-sum test. Bold values show statistically significant results.
(DOCX)

S6 Table Estimated sample coverage (Good’s Coverage), diversity richness (number of unique OTUs), and diversity index (Shannon) for 16S rRNA libraries. Samples IDs are coded as follows: experiment identifier (1, 2, or AL), sample day, substrate type, sample number (e.g. 1.2.Glass1 = Colonization Experiment #1, day 2, glass substrate, sample 1). Samples were normalized to 2,500 sequences to obtain equal sampling depths. A) Colonization Experiment #1, B) Colonization Experiment #2, and C) Environmental Samples.
(DOCX)

S1 Fig Hierarchical clustering dendrogram showing isolates’ relationship based on their antibiotic susceptibility profiles. ZOI data for each isolate was compared with that of all other isolates using Euclidean distance similarity, then clustered using a group average algorithm. Red symbols, Colonization Experiment #1; blue symbols, Colonization Experiment #2; green symbols, environmental samples. Plastic substrates (all types), triangles; glass substrate; +; water, filled circles.
(TIF)

S2 Fig Principal components analysis (PCA) of antibiotic profiles of *Vibrio* spp. (n = 97) by sample type. PC1 represents increasing susceptibility to streptomycin (S), rifampin (RA), and tetracycline (TE), and PC2 represents increasing susceptibility to chloramphenicol (C) and gentamicin (GM). Eigenvectors for each antibiotic are shown as lines adjacent to the corresponding labels. Symbols as in Fig 4. The PCA is overlain with Euclidean distance (value of 3) from the cluster analysis (Fig 4). Microplastics from environmental samples, hollow triangles; glass substrate, +; water, filled circles; polypropylene, filled triangles; polycarbonate, x; polystyrene, upside down filled triangles; high-density polyethylene, filled squares; low-density polyethylene, filled diamonds.
(TIF)

S3 Fig Principal components analysis (PCA) of antibiotic profiles identifying the 97 Vibrio isolates. PC1 and PC2 and symbols as in S2 Fig. Eigenvectors for each antibiotic are shown as lines. Isolates are color coded: magenta, *V*. *vulnificus*; blue, *V*. *parahaemolyticus*; green, *V*. *cholerae*.
(TIF)

## References

[1] Derraik JG. The pollution of the marine environment by plastic debris: a review. Marine pollution bulletin. 2002 9 1;44(9):842–52. 10.1016/s0025-326x(02)00220-5 12405208

[2] Lavers JL, Bond AL. Exceptional and rapid accumulation of anthropogenic debris on one of the world’s most remote and pristine islands. Proceedings of the National Academy of Sciences. 2017 6 6;114(23):6052–5. 10.1073/pnas.1619818114 PMC5468685 28507128

[3] Eriksen M, Lebreton LC, Carson HS, Thiel M, Moore CJ, Borerro JC, et al Plastic pollution in the world's oceans: more than 5 trillion plastic pieces weighing over 250,000 tons afloat at sea. PloS one. 2014 12 10;9(12):e111913 10.1371/journal.pone.0111913 25494041 PMC4262196

[4] Obbard RW, Sadri S, Wong YQ, Khitun AA, Baker I, Thompson RC. Global warming releases microplastic legacy frozen in Arctic Sea ice. Earth’s Future 2: 315–320.

[5] Kelly A, Lannuzel D, Rodemann T, Meiners KM, Auman HJ. Microplastic contamination in east Antarctic sea ice. Marine Pollution Bulletin. 2020 5 1;154:111130 10.1016/j.marpolbul.2020.111130 32319937

[6] Woodall LC, Sanchez-Vidal A, Canals M, Paterson GL, Coppock R, Sleight V, et al The deep sea is a major sink for microplastic debris. Royal Society open science. 2014 12 17;1(4):140317 10.1098/rsos.140317 26064573 PMC4448771

[7] Courtene-Jones W, Quinn B, Ewins C, Gary SF, Narayanaswamy BE. Microplastic accumulation in deep-sea sediments from the Rockall Trough. Marine Pollution Bulletin. 2020 5 1;154:111092 10.1016/j.marpolbul.2020.111092 32319921

[8] Carpenter EJ, Smith KL. Plastics on the Sargasso Sea surface. Science. 1972 3 17;175(4027):1240–1. 10.1126/science.175.4027.1240 5061243

[9] Colton JB, Knapp FD, Burns BR. Plastic particles in surface waters of the northwestern Atlantic. Science. 1974 8 9;185(4150):491–7. 10.1126/science.185.4150.491 17830390

[10] Barnes DK, Fraser KP. Rafting by five phyla on man-made flotsam in the Southern Ocean. Marine Ecology Progress Series. 2003 11 7;262:289–91.

[11] Masó M, Garcés E, Pagès F, Camp J. Drifting plastic debris as a potential vector for dispersing Harmful Algal Bloom (HAB) species. Scientia Marina. 2003 3 30;67(1):107–11.

[12] Reisser J, Shaw J, Hallegraeff G, Proietti M, Barnes DKA, Thums M, et al Millimeter-Sized Marine Plastics: A New Pelagic Habitat for Microorganisms and Invertebrates. PLoS ONE. 2014;9: e100289 10.1371/journal.pone.0100289 24941218 PMC4062529

[13] Debroas D, Mone A, Ter Halle A. Plastics in the North Atlantic garbage patch: a boat-microbe for hitchhikers and plastic degraders. Science of the Total Environment. 2017 12 1;599:1222–32. 10.1016/j.scitotenv.2017.05.059 28514840

[14] Zettler ER, Mincer TJ, Amaral-Zettler LA. Life in the “plastisphere”: microbial communities on plastic marine debris. Environmental science & technology. 2013 7 2;47(13):7137–46. 23745679 10.1021/es401288x

[15] De Tender CA, Devriese LI, Haegeman A, Maes S, Ruttink T, Dawyndt P. Bacterial community profiling of plastic litter in the Belgian part of the North Sea. Environmental science & technology. 2015 8 18;49(16):9629–38. 26204244 10.1021/acs.est.5b01093

[16] Amaral-Zettler LA, Zettler ER, Slikas B, Boyd GD, Melvin DW, Morrall CE, et al The biogeography of the Plastisphere: implications for policy. Frontiers in Ecology and the Environment. 2015 12;13(10):541–6.

[17] Kirstein IV, Kirmizi S, Wichels A, Garin-Fernandez A, Erler R, Löder M, et al Dangerous hitchhikers? Evidence for potentially pathogenic *Vibrio spp*. on microplastic particles. Marine environmental research. 2016 9 1;120:1–8. 10.1016/j.marenvres.2016.07.004 27411093

[18] Schmidt VT, Reveillaud J, Zettler E, Mincer TJ, Murphy L, Amaral-Zettler LA. Oligotyping reveals community level habitat selection within the genus Vibrio. Frontiers in microbiology. 2014 11 13;5:563 10.3389/fmicb.2014.00563 25431569 PMC4230168

[19] Froelich BA, Daines DA. In hot water: effects of climate change on Vibrio–human interactions. Environmental Microbiology. 2020 3 1 10.1111/1462-2920.14967 32114705

[20] Ashrafudoulla M, Mizan M, Rahaman F, Park H, Byun KH, Lee N, et al Genetic relationship, virulence factors, drug resistance profile and biofilm formation ability of *Vibrio parahaemolyticus* isolated from mussel. Frontiers in Microbiology. 2019 3 20;10:513 10.3389/fmicb.2019.00513 30949142 PMC6435529

[21] Warner EB, Oliver JD. Multiplex PCR assay for detection and simultaneous differentiation of genotypes of *Vibrio vulnificus* biotype 1. Foodborne pathogens and disease. 2008 10 1;5(5):691–3. 10.1089/fpd.2008.0120 18687035

[22] Nordstrom JL, Vickery MC, Blackstone GM, Murray SL, DePaola A. Development of a multiplex real-time PCR assay with an internal amplification control for the detection of total and pathogenic *Vibrio parahaemolyticus* bacteria in oysters. Applied and Environmental Microbiology. 2007 9 15;73(18):5840–7. 10.1128/AEM.00460-07 17644647 PMC2074920

[23] Fykse EM, Nilsen T, Nielsen AD, Tryland I, Delacroix S, Blatny JM. Real-time PCR and NASBA for rapid and sensitive detection of *Vibrio cholerae* in ballast water. Marine pollution bulletin. 2012 2 1;64(2):200–6. 10.1016/j.marpolbul.2011.12.007 22221710

[24] Wayne PA. National committee for clinical laboratory standards. Performance standards for antimicrobial disc susceptibility testing. 2002;12:01–53.

[25] Andrews JM. Determination of minimum inhibitory concentrations. Journal of antimicrobial Chemotherapy. 2001 7 1;48(suppl_1):5–16. 11420333 10.1093/jac/48.suppl_1.5

[26] Turner S, Pryer KM, Miao VP, Palmer JD. Investigating deep phylogenetic relationships among cyanobacteria and plastids by small subunit rRNA sequence analysis 1. Journal of Eukaryotic Microbiology. 1999 7;46(4):327–38. 10.1111/j.1550-7408.1999.tb04612.x 10461381

[27] Stackebrandt E, Goodfellow M. Nucleic acid techniques in bacterial systematics. Wiley; 1991.

[28] Schloss PD, Westcott SL, Ryabin T, Hall JR, Hartmann M, Hollister EB, et al Introducing mothur: open-source, platform-independent, community-supported software for describing and comparing microbial communities. Applied and environmental microbiology. 2009 12 1;75(23):7537–41. 10.1128/AEM.01541-09 19801464 PMC2786419

[29] Huse SM, Welch DM, Morrison HG, Sogin ML. Ironing out the wrinkles in the rare biosphere through improved OTU clustering. Environmental microbiology. 2010 7;12(7):1889–98. 10.1111/j.1462-2920.2010.02193.x 20236171 PMC2909393

[30] Rognes T, Flouri T, Nichols B, Quince C. Mah e, F.(2016). VSEARCH: A versatile open source tool for metagenomics. 10.7717/peerj.2584 PMC5075697 27781170

[31] Wang Q, Garrity GM, Tiedje JM. COLE JR(2007) Naïve Bayesian Classifier for rapid assignment of rRNA sequences into the new bacterial taxonomy. Appl. Environ. Microbiol.;73(16):5261–7. 10.1128/AEM.00062-07 17586664 PMC1950982

[32] Schloss PD, Westcott SL. Assessing and improving methods used in operational taxonomic unit-based approaches for 16S rRNA gene sequence analysis. Applied and environmental microbiology. 2011 5 15;77(10):3219–26. 10.1128/AEM.02810-10 21421784 PMC3126452

[33] Clarke KR, Gorley RN. Primer. PRIMER-e, Plymouth. 2006.

[34] Yonkos LT, Friedel EA, Perez-Reyes AC, Ghosal S, Arthur CD. Microplastics in four estuarine rivers in the Chesapeake Bay, USA. Environmental science & technology. 2014 12 16;48(24):14195–202. 25389665 10.1021/es5036317

[35] Bikker J, Lawson J, Wilson S, Rochman CM. Microplastics and other anthropogenic particles in the surface waters of the Chesapeake Bay. Marine Pollution Bulletin. 2020 7 1;156:111257 10.1016/j.marpolbul.2020.111257 32510399

[36] Enders K, Lenz R, Stedmon CA, Nielsen TG. Abundance, size and polymer composition of marine microplastics≥ 10 μm in the Atlantic Ocean and their modelled vertical distribution. Marine pollution bulletin. 2015 11 15;100(1):70–81. 10.1016/j.marpolbul.2015.09.027 26454631

[37] Morét-Ferguson S, Law KL, Proskurowski G, Murphy EK, Peacock EE, Reddy CM. The size, mass, and composition of plastic debris in the western North Atlantic Ocean. Marine Pollution Bulletin. 2010 10 1;60(10):1873–8. 10.1016/j.marpolbul.2010.07.020 20709339

[38] Geyer R, Jambeck JR, Law KL. Production, use, and fate of all plastics ever made. Science advances. 2017 7 1;3(7):e1700782 10.1126/sciadv.1700782 28776036 PMC5517107

[39] Oberbeckmann S, Loeder MG, Gerdts G, Osborn AM. Spatial and seasonal variation in diversity and structure of microbial biofilms on marine plastics in Northern European waters. FEMS microbiology ecology. 2014 11 1;90(2):478–92. 10.1111/1574-6941.12409 25109340

[40] Bryant JA, Clemente TM, Viviani DA, Fong AA, Thomas KA, Kemp P, et al Diversity and activity of communities inhabiting plastic debris in the North Pacific Gyre. MSystems. 2016 6 28;1(3):e00024–16. 10.1128/mSystems.00024-16 27822538 PMC5069773

[41] Oberbeckmann S, Kreikemeyer B, Labrenz M. Environmental factors support the formation of specific bacterial assemblages on microplastics. Frontiers in Microbiology. 2018 1 19;8:2709 10.3389/fmicb.2017.02709 29403454 PMC5785724

[42] Oberbeckmann S, Osborn AM, Duhaime MB. Microbes on a bottle: substrate, season and geography influence community composition of microbes colonizing marine plastic debris. PLoS One. 2016 8 3;11(8):e0159289 10.1371/journal.pone.0159289 27487037 PMC4972250

[43] Lyons MM, Ward JE, Gaff H, Hicks RE, Drake JM, Dobbs FC. Theory of island biogeography on a microscopic scale: organic aggregates as islands for aquatic pathogens. Aquatic Microbial Ecology. 2010 5 4;60(1):1–3.

[44] Kelly MT. Effect of temperature and salinity on *Vibrio (Beneckea) vulnificus* occurrence in a Gulf Coast environment. Applied and environmental microbiology. 1982 10 1;44(4):820–4. 10.1128/AEM.44.4.820-824.1982 7149714 PMC242103

[45] Huq A, Haley BJ, Taviani E, Chen A, Hasan NA, Colwell RR. Detection, isolation, and identification of *Vibrio cholerae* from the environment. Current protocols in microbiology. 2012 8;26(1):6A–5. 10.1002/9780471729259.mc06a05s26 PMC3461827 22875567

[46] Arias-Andres M, Klümper U, Rojas-Jimenez K, Grossart HP. Microplastic pollution increases gene exchange in aquatic ecosystems. Environmental Pollution. 2018 6 1;237:253–61. 10.1016/j.envpol.2018.02.058 29494919

[47] Ahmed AM, Motoi Y, Sato M, Maruyama A, Watanabe H, Fukumoto Y, Shimamoto T. Zoo animals as reservoirs of gram-negative bacteria harboring integrons and antimicrobial resistance genes. Applied and environmental microbiology. 2007 10 15;73(20):6686–90. 10.1128/AEM.01054-07 17720829 PMC2075039

[48] Suzuki M, Nakagawa Y, Harayama S, Yamamoto S. Phylogenetic analysis and taxonomic study of marine Cytophaga-like bacteria: proposal for *Tenacibaculum* gen. nov. with *Tenacibaculum maritimum* comb. nov. and *Tenacibaculum ovolyticum* comb. nov., and description of *Tenacibaculum mesophilum* sp. nov. and *Tenacibaculum amylolyticum* sp. nov. International journal of systematic and evolutionary microbiology. 2001 9 1;51(5):1639–52. 11594591 10.1099/00207713-51-5-1639

