# Supplementary material for: Bacterial biofilms colonizing plastics in estuarine waters, with an emphasis on Vibrio spp. and their antibacterial resistance
Source: PLoS One. 2020 Aug 17;15(8):e0237704. doi: 10.1371/journal.pone.0237704 (PMC7430737; doi:10.1371/journal.pone.0237704)
Supplement: S1 Table — Temperature (°C) and salinity (ppt) values for A) Colonization Experiment #1, B) Colonization Experiment #2, and C) Environmental Samples. (DOCX) [file pone.0237704.s001.docx]

**S1 Table.** Temperature (°C) and salinity (ppt) values for A) Colonization Experiment #1, B) Colonization Experiment #2, and C) Environmental Samples.

A) Colonization Experiment #1

| Sample date (day) | Temperature (°C) | Salinity (ppt) |
| --- | --- | --- |
| 1 | 21.5 | 19 |
| 2 | 21.0 | 19 |
| 4 | 20.3 | 19 |
| 8 | 14.8 | 20 |
| 16 | 16.8 | 22 |
| 30 | 18.0 | 21 |

B) Colonization Experiment #2

| Sample date | Temperature | Salinity |
| --- | --- | --- |
| 1 | 9.5 | 18 |
| 2 | 6.5 | 18 |
| 4 | 7.4 | 17 |
| 9 | 2.1 | 18 |
| 17 | 5.2 | 15 |
| 31 | 5.2 | 12 |

C) Environmental Samples

| Sample date | Temperature | Salinity |
| --- | --- | --- |
| June 2, 2015 | 24.9 | - |
| June 3, 2015 | 22.3 | - |
| June 4, 2015 | 22.1 | - |
| June 9, 2015 | 25.6 | - |
| June 15, 2015 | 28.2 | - |
| June 18, 2015 | 28.7 | - |
| July 1, 2015 | 26.9 | - |
| August 13, 2015 | 27.0 | - |
| August 20, 2015 | 30.1 | - |
| September 2, 2015 | 26.0 | - |
| September 11, 2015 | 24.5 | 23 |
| October 29, 2015 | 18.9 | 22 |
| November 9, 2015 | 16.1 | 21 |
| November 24, 2015 | 10.3 | 17 |
| November 25, 2015 | 12.1 | 18 |
