# Supplementary material for: Bacterial biofilms colonizing plastics in estuarine waters, with an emphasis on Vibrio spp. and their antibacterial resistance
Source: PLoS One. 2020 Aug 17;15(8):e0237704. doi: 10.1371/journal.pone.0237704 (PMC7430737; doi:10.1371/journal.pone.0237704)
Supplement: S2 Table — Columns represent substrates (LDPE, HDPE, PP, PC, Glass) and rows represent days (2, 4, 16, 30). (DOCX) [file pone.0237704.s002.docx]

**S2 Table.** ANOVA on concentration of putative *Vibrio* spp. in Colonization Experiment #1. Columns represent substrates (LDPE, HDPE, PP, PC, Glass) and rows represent days (2, 4, 16, 30).

| Source | SS | df | MS | F | Prob>F |
| --- | --- | --- | --- | --- | --- |
| Columns | 0.404 | 4 | 0.101 | 0.593 | 0.670 |
| Rows | 82.623 | 3 | 27.541 | 161.653 | <0.001 |
| Interaction | 1.658 | 12 | 0.138 | 0.8101 | 0.638 |
| Error | 5.963 | 35 | 0.170 |  |  |
| Total | 90.647 | 54 |  |  |  |
