# Supplementary material for: Bacterial biofilms colonizing plastics in estuarine waters, with an emphasis on Vibrio spp. and their antibacterial resistance
Source: PLoS One. 2020 Aug 17;15(8):e0237704. doi: 10.1371/journal.pone.0237704 (PMC7430737; doi:10.1371/journal.pone.0237704)
Supplement: S3 Table — Columns represent substrates (LDPE, HDPE, PP, PC, Glass, and PS) and rows represent days (2, 4, 9, 17, 31). (DOCX) [file pone.0237704.s003.docx]

**S3 Table**. ANOVA on concentration of putative *Vibrio* spp. in Colonization Experiment #2. Columns represent substrates (LDPE, HDPE, PP, PC, Glass, and PS) and rows represent days (2, 4, 9, 17, 31).

| Source | SS | df | MS | F | Prob>F |
| --- | --- | --- | --- | --- | --- |
| Columns | 1.125 | 5 | 0.225 | 6.258 | <0.001 |
| Rows | 30.096 | 4 | 7.524 | 209.345 | <0.001 |
| Interaction | 3.852 | 20 | 0.193 | 5.359 | <0.001 |
| Error | 1.977 | 55 | 0.036 |  |  |
| Total | 37.049 | 84 |  |  |  |
