# Supplementary material for: Bacterial biofilms colonizing plastics in estuarine waters, with an emphasis on Vibrio spp. and their antibacterial resistance
Source: PLoS One. 2020 Aug 17;15(8):e0237704. doi: 10.1371/journal.pone.0237704 (PMC7430737; doi:10.1371/journal.pone.0237704)
Supplement: S4 Table — The magnitude of loading, positive or negative, indicates the degree of influence the antibiotic has on each principal component. Loadings for the first three PCs are shown and for each, loadings having high absolute values are bolded. (DOCX) [file pone.0237704.s004.docx]

**S4 Table.** Loading values from principal component analysis of antibiotic susceptibility data for six antibiotics (abbreviated as in Table 2). The magnitude of loading, positive or negative, indicates the degree of influence the antibiotic has on each principal component. Loadings for the first three PCs are shown and for each, loadings having high absolute values are bolded.

| Antibiotics | PC1 | PC2 | PC3 | Antibiotics | PC1 | PC2 | PC3 |
| --- | --- | --- | --- | --- | --- | --- | --- |
| AM | 0.36 | -0.02 | **0.92** | RA | **0.88** | 0.25 | -0.11 |
| GM | 0.39 | **-0.79** | -0.22 | C | -0.34 | **-0.82** | 0.17 |
| S | **0.83** | -0.12 | 0.04 | TE | **0.92** | -0.09 | -0.14 |
