# Supplementary material for: Bacterial biofilms colonizing plastics in estuarine waters, with an emphasis on Vibrio spp. and their antibacterial resistance
Source: PLoS One. 2020 Aug 17;15(8):e0237704. doi: 10.1371/journal.pone.0237704 (PMC7430737; doi:10.1371/journal.pone.0237704)
Supplement: S5 Table — Bold values show statistically significant results. (DOCX) [file pone.0237704.s005.docx]

**S5 Table.** Comparisons of Zones of Inhibition (ZOI) for six antibiotics (abbreviated as in Table 2) in isolates from colonization experiments versus isolates from environmental samples using a Wilcoxon rank-sum test. Bold values show statistically significant results.

| Colonization Experiment #1 vs. Environmental Samples; (n=55) | | | Colonization Experiment #2 vs. Environmental Samples; (n=48) | | |
| --- | --- | --- | --- | --- | --- |
| Antibiotic | Statistic (Z) | P-value | Antibiotic | Statistic (Z) | P-value |
| AM | -0.91 | 0.36 | AM | 0.52 | 0.60 |
| GM | -3.68 | **2.33E-04** | GM | 0.26 | 0.79 |
| S | -4.54 | **5.53E-06** | S | -2.33 | **0.02** |
| RA | 1.08 | 0.28 | RA | -0.68 | 0.50 |
| C | -2.27 | **0.02** | C | -0.12 | 0.91 |
| TE | -1.97 | **0.05** | TE | 0.26 | 0.79 |
