# Supplementary material for: Bacterial biofilms colonizing plastics in estuarine waters, with an emphasis on Vibrio spp. and their antibacterial resistance
Source: PLoS One. 2020 Aug 17;15(8):e0237704. doi: 10.1371/journal.pone.0237704 (PMC7430737; doi:10.1371/journal.pone.0237704)
Supplement: S6 Table — Samples IDs are coded as follows: experiment identifier (1, 2, or AL), sample day, substrate type, sample number (e.g. 1.2.Glass1 = Colonization Experiment #1, day 2, glass substrate, sample 1). Samples were normalized to 2,500 sequences to obtain equal sampling depths. A) Colonization Experiment #1, B) Colonization Experiment #2, and C) Environmental Samples. (DOCX) [file pone.0237704.s006.docx]

**S6 Table.** Estimated sample coverage (Good’s Coverage), diversity richness (number of unique OTUs), and diversity index (Shannon) for 16S rRNA libraries. Samples IDs are coded as follows: experiment identifier (1, 2, or AL), sample day, substrate type, sample number (e.g. 1.2.Glass1 = Colonization Experiment #1, day 2, glass substrate, sample 1). Samples were normalized to 2,500 sequences to obtain equal sampling depths. A) Colonization Experiment #1, B) Colonization Experiment #2, and C) Environmental Samples.

A) Colonization Experiment #1

| **Sample ID** | **Good's Coverage** | **# of Unique OTUs** | **Shannon** |
| --- | --- | --- | --- |
| 1.2.Glass1 | 0.993 | 56 | 2.28 |
| 1.2.Glass2 | 0.992 | 95 | 2.71 |
| 1.2.HDPE1 | 0.993 | 120 | 3.25 |
| 1.2.HDPE2 | 0.991 | 104 | 2.96 |
| 1.2.LDPE1 | 0.970 | 201 | 3.49 |
| 1.2.LDPE2 | 0.991 | 98 | 3.39 |
| 1.2.PC1 | 0.992 | 100 | 2.79 |
| 1.2.PC2 | 0.992 | 108 | 2.43 |
| 1.2.PP1 | 0.992 | 83 | 2.95 |
| 1.2.PP2 | 0.992 | 115 | 3.21 |
| 1.4.Glass1 | 0.988 | 165 | 3.26 |
| 1.4.Glass2 | 0.983 | 187 | 2.51 |
| 1.4.Glass3 | 0.981 | 204 | 2.69 |
| 1.4.HDPE1 | 0.981 | 206 | 2.86 |
| 1.4.HDPE2 | 0.984 | 181 | 2.54 |
| 1.4.HDPE3 | 0.981 | 198 | 2.49 |
| 1.4.LDPE1 | 0.983 | 171 | 2.91 |
| 1.4.LDPE2 | 0.989 | 142 | 3.02 |
| 1.4.LDPE3 | 0.985 | 158 | 2.44 |
| 1.4.PC1 | 0.975 | 222 | 3.4 |
| 1.4.PC2 | 0.980 | 209 | 3.12 |
| 1.4.PC3 | 0.979 | 208 | 3.17 |
| 1.4.PP1 | 0.979 | 224 | 3.28 |
| 1.4.PP2 | 0.981 | 217 | 3.18 |
| 1.4.PP3 | 0.980 | 214 | 3.28 |
| 1.16.Glass1 | 0.982 | 181 | 1.69 |
| 1.16.Glass2 | 0.980 | 178 | 1.82 |
| 1.16.HDPE1 | 0.979 | 209 | 2.3 |
| 1.16.HDPE2 | 0.977 | 218 | 2.95 |
| 1.16.LDPE1 | 0.979 | 203 | 2.3 |
| 1.16.LDPE2 | 0.981 | 185 | 2.21 |
| 1.16.PC1 | 0.980 | 193 | 2.26 |
| 1.16.PC2 | 0.981 | 199 | 2.37 |
| 1.16.PP1 | 0.979 | 204 | 3.16 |
| 1.16.PP2 | 0.979 | 203 | 3.16 |
| 1.30.Glass1 | 0.977 | 219 | 2.96 |
| 1.30.Glass2 | 0.978 | 210 | 3.25 |
| 1.30.Glass3 | 0.980 | 218 | 3.45 |
| 1.30.HDPE1 | 0.978 | 215 | 3.11 |
| 1.30.HDPE2 | 0.972 | 201 | 3.39 |
| 1.30.HDPE3 | 0.976 | 228 | 3.57 |
| 1.30.LDPE1 | 0.975 | 213 | 3 |
| 1.30.LDPE2 | 0.978 | 198 | 2.86 |
| 1.30.LDPE3 | 0.977 | 214 | 3.71 |
| 1.30.PC1 | 0.976 | 253 | 3.67 |
| 1.30.PC2 | 0.977 | 221 | 3.63 |
| 1.30.PC3 | 0.978 | 216 | 2.99 |
| 1.30.PP1 | 0.978 | 228 | 3.67 |
| 1.30.PP2 | 0.973 | 226 | 3.6 |
| 1.30.PP3 | 0.975 | 256 | 3.55 |

B) Colonization Experiment #2

| **Sample ID** | **Good's Coverage** | **# of Unique OTUs** | **Shannon** |
| --- | --- | --- | --- |
| 2.2.Glass1&2 | 0.993 | 132 | 2.26 |
| 2.2.Glass3 | 0.992 | 67 | 2.32 |
| 2.2.H2O1 | 0.988 | 118 | 2.72 |
| 2.2.H2O2 | 0.988 | 120 | 2.7 |
| 2.2.HDPE1 | 0.989 | 161 | 3.43 |
| 2.2.HDPE2 | 0.995 | 86 | 3.57 |
| 2.2.LDPE1 | 0.993 | 140 | 2.97 |
| 2.2.LDPE2 | 0.994 | 109 | 2.53 |
| 2.2.PC1 | 0.994 | 125 | 3.02 |
| 2.2.PC2 | 0.989 | 150 | 3.23 |
| 2.2.PS1 | 0.993 | 66 | 2.37 |
| 2.2.PS2 | 0.992 | 126 | 3.26 |
| 2.2.PP1 | 0.993 | 126 | 3.7 |
| 2.2.PP2 | 0.990 | 130 | 3.74 |
| 2.4.Glass1 | 0.993 | 104 | 2.26 |
| 2.4.Glass2 | 0.993 | 104 | 2.17 |
| 2.4.Glass3 | 0.993 | 86 | 2.03 |
| 2.4.H2O1 | 0.988 | 113 | 2.71 |
| 2.4.H2O2 | 0.987 | 112 | 2.7 |
| 2.4.H2O3 | 0.989 | 106 | 2.65 |
| 2.4.HDPE1 | 0.986 | 165 | 1.77 |
| 2.4.HDPE2 | 0.991 | 128 | 2.04 |
| 2.4.HDPE3 | 0.991 | 128 | 1.92 |
| 2.4.LDPE1 | 0.981 | 189 | 1.79 |
| 2.4.LDPE2 | 0.986 | 160 | 2.41 |
| 2.4.LDPE3 | 0.986 | 164 | 1.52 |
| 2.4.PC1 | 0.994 | 99 | 2.29 |
| 2.4.PC2 | 0.984 | 167 | 2.05 |
| 2.4.PC3 | 0.983 | 163 | 1.79 |
| 2.4.PS1 | 0.992 | 85 | 1.2 |
| 2.4.PS2 | 0.987 | 117 | 1.19 |
| 2.4.PS3 | 0.991 | 122 | 2.06 |
| 2.4.PP1 | 0.993 | 109 | 2.01 |
| 2.4.PP2 | 0.986 | 147 | 1.82 |
| 2.4.PP3 | 0.977 | 202 | 2.72 |
| 2.17.Glass1 | 0.973 | 293 | 2.35 |
| 2.17.Glass2 | 0.974 | 187 | 2.18 |
| 2.17.H2O1 | 0.979 | 192 | 2.39 |
| 2.17.H2O2 | 0.987 | 132 | 2.26 |
| 2.17.HDPE1 | 0.978 | 231 | 1.54 |
| 2.17.HDPE2 | 0.978 | 221 | 2.3 |
| 2.17.LDPE1 | 0.978 | 209 | 1.81 |
| 2.17.LDPE2 | 0.975 | 197 | 2.11 |
| 2.17.PC1 | 0.976 | 203 | 1.6 |
| 2.17.PC2 | 0.973 | 326 | 2.57 |
| 2.17.PS1 | 0.979 | 191 | 1.59 |
| 2.17.PS2 | 0.980 | 209 | 1.55 |
| 2.17.PP1 | 0.974 | 215 | 2.03 |
| 2.17.PP2 | 0.977 | 213 | 1.85 |
| 2.31.Glass1 | 0.976 | 219 | 2.27 |
| 2.31.Glass2 | 0.978 | 199 | 2.3 |
| 2.31.Glass3 | 0.974 | 233 | 2.58 |
| 2.31.H2O1 | 0.985 | 162 | 2.98 |
| 2.31.H2O2 | 0.984 | 154 | 2.96 |
| 2.31.H2O3 | 0.985 | 160 | 2.91 |
| 2.31.HDPE1 | 0.975 | 235 | 2.64 |
| 2.31.HDPE2 | 0.980 | 203 | 2.47 |
| 2.31.HDPE3 | 0.977 | 231 | 2.44 |
| 2.31.LDPE1 | 0.975 | 210 | 2.8 |
| 2.31.LDPE2 | 0.973 | 232 | 2.9 |
| 2.31.LDPE3 | 0.975 | 189 | 2.78 |
| 2.31.PC1 | 0.977 | 193 | 2.64 |
| 2.31.PC2 | 0.973 | 219 | 2.85 |
| 2.31.PC3 | 0.973 | 223 | 2.98 |
| 2.31.PS1 | 0.976 | 236 | 2.5 |
| 2.31.PS2 | 0.976 | 215 | 2.6 |
| 2.31.PS3 | 0.970 | 216 | 3.06 |
| 2.31.PP1 | 0.973 | 240 | 2.62 |
| 2.31.PP2 | 0.976 | 214 | 2.59 |
| 2.31.PP3 | 0.977 | 209 | 2.76 |

C) Environmental Samples

| **Sample ID** | **Good's Coverage** | **# of Unique OTUs** | **Shannon** |
| --- | --- | --- | --- |
| AL38 | 0.991 | 134 | 3.43 |
| AL38.H2O | 0.985 | 141 | 2.72 |
| AL42 | 0.979 | 211 | 2.91 |
| AL42.H2O1 | 0.986 | 136 | 2.88 |
| AL43 | 0.989 | 97 | 3.4 |
| AL43.H2O2 | 0.988 | 123 | 3 |
| AL46 | 0.979 | 203 | 3.18 |
| AL46.H2O1 | 0.987 | 127 | 2.8 |
| AL46.H2O2 | 0.985 | 141 | 2.92 |
