# Supplementary figures and images for: Bacterial biofilms colonizing plastics in estuarine waters, with an emphasis on Vibrio spp. and their antibacterial resistance
Source: PLoS One. 2020 Aug 17;15(8):e0237704. doi: 10.1371/journal.pone.0237704 (PMC7430737; doi:10.1371/journal.pone.0237704)

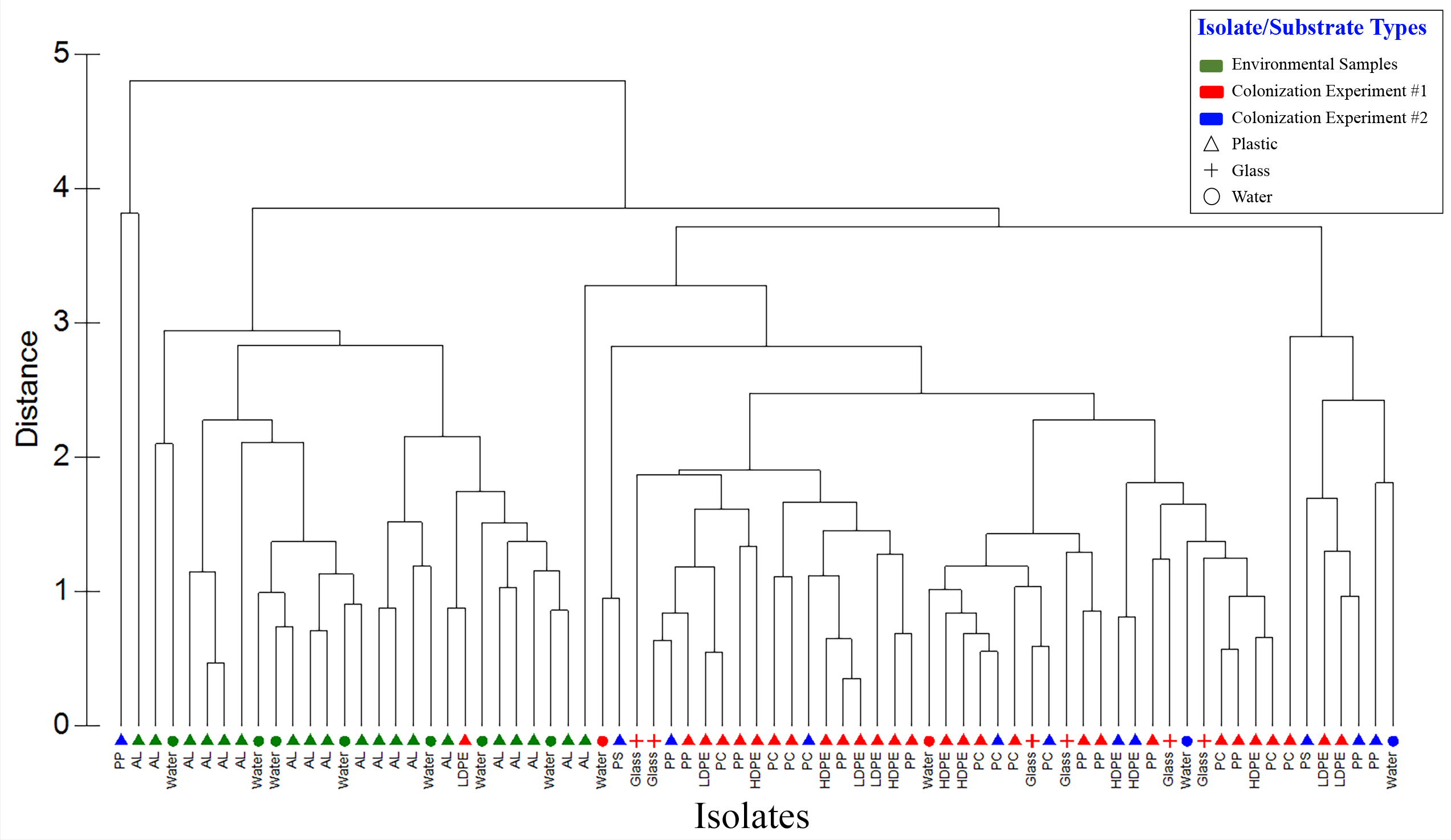

Supplement: S1 Fig — ZOI data for each isolate was compared with that of all other isolates using Euclidean distance similarity, then clustered using a group average algorithm. Red symbols, Colonization Experiment #1; blue symbols, Colonization Experiment #2; green symbols, environmental samples. Plastic substrates (all types), triangles; glass substrate; +; water, filled circles. (TIF) [file pone.0237704.s007.tif]

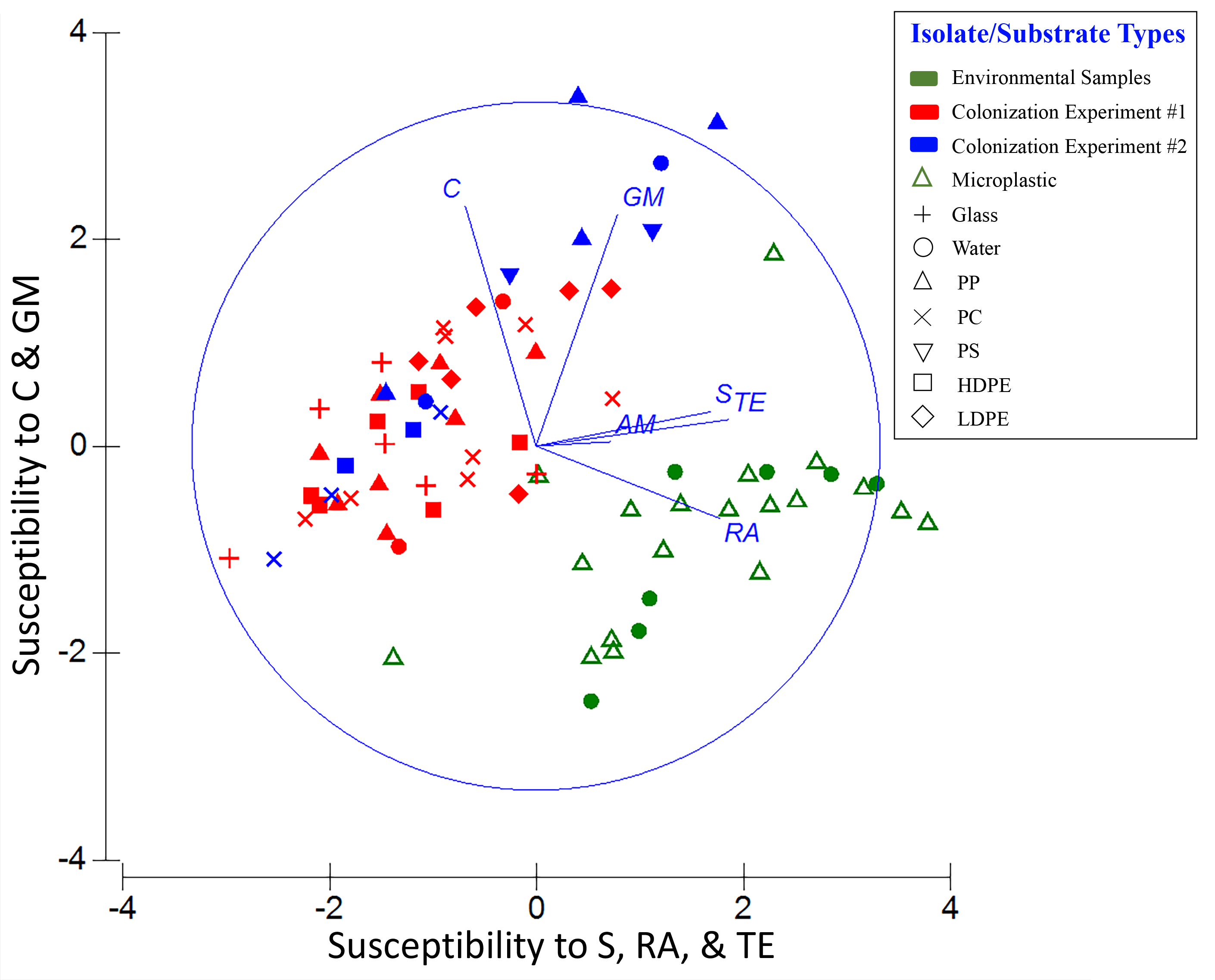

Supplement: S2 Fig — PC1 represents increasing susceptibility to streptomycin (S), rifampin (RA), and tetracycline (TE), and PC2 represents increasing susceptibility to chloramphenicol (C) and gentamicin (GM). Eigenvectors for each antibiotic are shown as lines adjacent to the corresponding labels. Symbols as in Fig 4. The PCA is overlain with Euclidean distance (value of 3) from the cluster analysis (Fig 4). Microplastics from environmental samples, hollow triangles; glass substrate, +; water, filled circles; polypropylene, filled triangles; polycarbonate, x; polystyrene, upside down filled triangles; high-density polyethylene, filled squares; low-density polyethylene, filled diamonds. (TIF) [file pone.0237704.s008.tif]

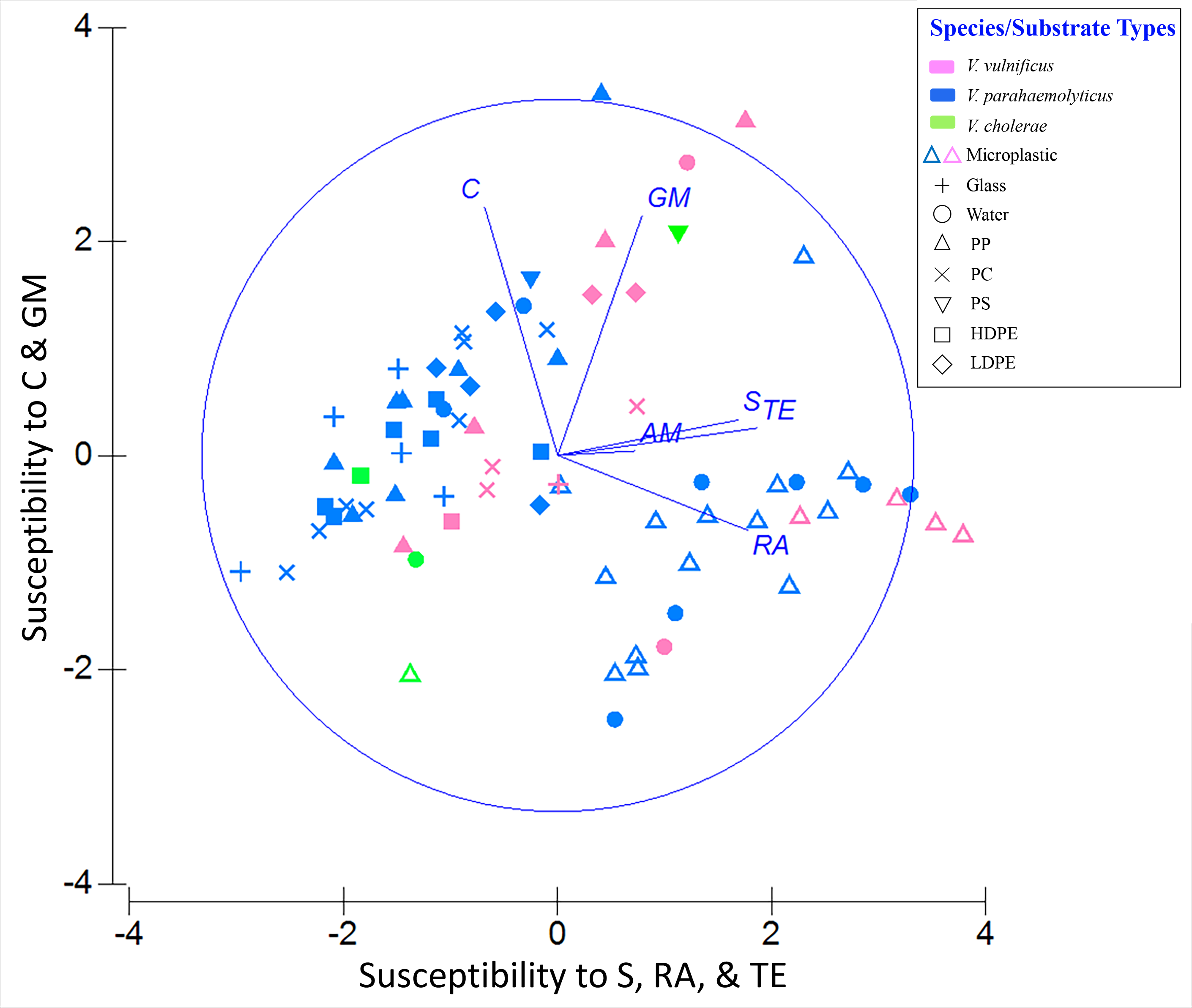

Supplement: S3 Fig — PC1 and PC2 and symbols as in S2 Fig. Eigenvectors for each antibiotic are shown as lines. Isolates are color coded: magenta, V. vulnificus; blue, V. parahaemolyticus; green, V. cholerae. (TIF) [file pone.0237704.s009.tif]
